# Safety and immunogenicity of inactivated poliovirus vaccine schedules for the post-eradication era: a randomised open-label, multicentre, phase 3, non-inferiority trial

**DOI:** 10.1016/S1473-3099(20)30555-7

**Published:** 2021-04

**Authors:** Ananda S Bandyopadhyay, Chris Gast, Luis Rivera, Xavier Sáez-Llorens, M Steven Oberste, William C Weldon, John Modlin, Ralf Clemens, Sue Ann Costa Clemens, Jose Jimeno, Ricardo Rüttimann

**Affiliations:** a Polio, Global Development, Bill & Melinda Gates Foundation, Seattle, USA; b Biostatistics Consultant, Seattle, Washington, USA; c Hospital Maternidad Nuestra Señora de la Altagracia, Santo Domingo, Dominican Republic; d Department of Infectious Disease, Hospital del Niño Dr José Renán Esquivel, Panama City, Panama; e Division of Viral Diseases, National Center for Immunization and Respiratory Diseases, CDC, Atlanta, GA, USA; f Global Research in Infectious Diseases, Rio de Janeiro, Brazil; g Fighting Infectious Diseases in Emerging Countries, Miami, FL, USA

## Abstract

**Background:**

Following the global eradication of wild poliovirus, countries using live attenuated oral poliovirus vaccines will transition to exclusive use of inactivated poliovirus vaccine (IPV) or fractional doses of IPV (f-IPV; a f-IPV dose is one-fifth of a normal IPV dose), but IPV supply and cost constraints will necessitate dose-sparing strategies. We compared immunisation schedules of f-IPV and IPV to inform the choice of optimal post-eradication schedule.

**Methods:**

This randomised open-label, multicentre, phase 3, non-inferiority trial was done at two centres in Panama and one in the Dominican Republic. Eligible participants were healthy 6-week-old infants with no signs of febrile illness or known allergy to vaccine components. Infants were randomly assigned (1:1:1:1, 1:1:1:2, 2:1:1:1), using computer-generated blocks of four or five until the groups were full, to one of four groups and received: two doses of intradermal f-IPV (administered at 14 and 36 weeks; two f-IPV group); or three doses of intradermal f-IPV (administered at 10, 14, and 36 weeks; three f-IPV group); or two doses of intramuscular IPV (administered at 14 and 36 weeks; two IPV group); or three doses of intramuscular IPV (administered at 10, 14, and 36 weeks; three IPV group). The primary outcome was seroconversion rates based on neutralising antibodies for poliovirus type 1 and type 2 at baseline and at 40 weeks (4 weeks after the second or third vaccinations) in the per-protocol population to allow non-inferiority and eventually superiority comparisons between vaccines and regimens. Three co-primary outcomes concerning poliovirus types 1 and 2 were to determine if seroconversion rates at 40 weeks of age after a two-dose regimen (administered at weeks 14 and 36) of intradermally administered f-IPV were non-inferior to a corresponding two-dose regimen of intramuscular IPV; if seroconversion rates at 40 weeks of age after a two-dose IPV regimen (weeks 14 and 36) were non-inferior to those after a three-dose IPV regimen (weeks 10, 14, and 36); and if seroconversion rates after a two-dose f-IPV regimen (weeks 14 and 36) were non-inferior to those after a three-dose f-IPV regimen (weeks 10, 14, and 36). The non-inferiority boundary was set at −10% for the lower bound of the two-sided 95% CI for the seroconversion rate difference.. Safety was assessed as serious adverse events and important medical events. This study is registered on ClinicalTrials.gov, NCT03239496.

**Findings:**

From Oct 23, 2017, to Nov 13, 2018, we enrolled 773 infants (372 [48%] girls) in Panama and the Dominican Republic (two f-IPV group n=217, three f-IPV group n=178, two IPV group n=178, and three IPV group n=200). 686 infants received all scheduled vaccine doses and were included in the per-protocol analysis. We observed non-inferiority for poliovirus type 1 seroconversion rate at 40 weeks for the two f-IPV dose schedule (95·9% [95% CI 92·0–98·2]) versus the two IPV dose schedule (98·7% [95·4–99·8]), and for the three f-IPV dose schedule (98·8% [95·6–99·8]) versus the three IPV dose schedule (100% [97·9–100]). Similarly, poliovirus type 2 seroconversion rate at 40 weeks for the two f-IPV dose schedule (97·9% [94·8–99·4]) versus the two IPV dose schedule (99·4% [96·4–100]), and for the three f-IPV dose schedule (100% [97·7–100]) versus the three IPV dose schedule (100% [97·9–100]) were non-inferior. Seroconversion rate for the two f-IPV regimen was statistically superior 4 weeks after the last vaccine dose in the 14 and 36 week schedule (95·9% [92·0–98·2]) compared with the 10 and 14 week schedule (83·2% [76·5–88·6]; p=0·0062) for poliovirus type 1. Statistical superiority of the 14 and 36 week schedule was also found for poliovirus type 2 (14 and 36 week schedule 97·9% [94·8–99·4] *vs* 10 and 14 week schedule 83·9% [77·2–89·2]; p=0·0062), and poliovirus type 3 (14 and 36 week schedule 84·5% [78·7–89·3] *vs* 10 and 14 week schedule 73·3% [65·8–79·9]; p=0·0062). For IPV, a two dose regimen administered at 14 and 36 weeks (99·4% [96·4–100]) was superior a 10 and 14 week schedule (88·9% [83·4–93·1]; p<0·0001) for poliovirus type 2, but not for type 1 (14 and 36 week schedule 98·7% [95·4–99·8] *vs* 10 and 14 week schedule 95·6% [91·4–98·1]), or type 3 (14 and 36 week schedule 97·4% [93·5–99·3] *vs* 10 and 14 week schedule 93·9% [89·3–96·9]). There were no related serious adverse events or important medical events reported in any group showing safety was unaffected by administration route or schedule.

**Interpretation:**

Our observations suggest that adequate immunity against poliovirus type 1 and type 2 is provided by two doses of either IPV or f-IPV at 14 and 36 weeks of age, and broad immunity is provided with three doses of f-IPV, enabling substantial savings in cost and supply. These novel clinical data will inform global polio immunisation policy for the post-eradication era.

**Funding:**

Bill & Melinda Gates Foundation.

## Introduction

For decades, trivalent oral poliovirus vaccines (OPV; poliovirus type 1, type 2, and type 3) were the preferred vaccines for polio control and eradication, with trivalent inactivated poliovirus vaccine (IPV) exclusively used in a few high-income countries until 2000.^1^ Use of these vaccines has driven the progress toward global eradication of wild polioviruses.^2^ Eradication of wild poliovirus type 2 was declared on Sept 20, 2015, with the last reported case in October 1999, and the eradication of wild poliovirus type 3 was declared on Oct 17, 2019, following the last reported case in November, 2012. Since 2017, wild poliovirus type 1 cases have only been reported in Afghanistan (14 cases in 2017, 21 cases in 2018, 29 cases in 2019, and 47 cases to date in 2020) and Pakistan (eight cases in 2017, 12 cases in 2018, 147 cases in 2019, and 73 cases to date in 2020).^3^

After the certification of global wild poliovirus type 2 eradication, the WHO Strategic Advisory Group of Experts on Immunisation (SAGE) recommended global withdrawal of live type 2 poliovirus vaccines from all use from May, 2016, replacing trivalent OPV with bivalent OPV (types 1 and 3), and the inclusion of at least one dose of IPV in routine infant schedules. This dose of IPV is the only source of immunity against poliovirus type 2; it is administered to prevent paralysis because of the risks associated with circulating vaccine-derived polioviruses.^3^, ^4^ Areas with low vaccination coverage, where homotypic wild polioviruses have been eliminated, with epidemiologic conditions—such as low socioeconomic status, poor hygiene and sanitation, and high population density—that favour poliovirus transmission are at increased risk of circulating vaccine-derived poliovirus outbreaks caused by reverted strains of live attenuated vaccine virus that have regained transmissibility and neurovirulence.^5^ Since the global cessation of routine use of poliovirus type 2 Sabin OPV, an increasing number of circulating vaccine-derived poliovirus type 2 outbreaks have been reported. The number of reported circulating vaccine-derived poliovirus outbreaks more than tripled from nine between Jan 1, 2017 and June 30, 2018 to 29 between Jan 1, 2018 and June 30, 2019.^6^ This has led the WHO to designate these outbreaks as a Public Health Emergency of International Concern.

**Research in context**

**Evidence before this study**

The declaration of eradication of wild poliovirus type 3 on October 17, 2019 means only the continuing circulation of wild poliovirus type 1 in Afghanistan and Pakistan confounds the goal of global eradication of wild polioviruses. Once eradication is achieved current planning suggests that children will still need to be immunised against polio for at least another decade primarily because of the ongoing threat posed by circulating vaccine-derived polioviruses. To accomplish this strategy while removing the source of circulating vaccine-derived polioviruses will require the replacement of all routine use of oral poliovirus vaccines (OPV) with inactivated poliovirus vaccines (IPV). Because the manufacturing capacity of IPV is currently inadequate for projected global demand and their cost makes them unaffordable for many low-income countries, WHO and associated bodies have recommended use of fewer doses or fractional doses of IPV (f-IPV) administered intradermally.

We searched PubMed from the inception of the database until April 27, 2020, using the terms “polio”, “vaccine”, “fractional”, and “immunogenicity”. We found 20 reviews and clinical studies in which f-IPV was used as priming or booster doses, but few of these were clinical studies in which f-IPV was used for the full infant primary immunisation series.

**Added value of this study**

The present study was done specifically to investigate the responses to two or three doses of f-IPV in polio vaccine-naive infants as primary vaccinations, and to directly compare these with equivalent schedules using full dose IPV. By using a delayed schedule we were also able to assess the effect of the timing of these vaccinations on the immune responses, and the influence of pre-existing maternal antibodies on the final immunogenicity.

**Implications of all the available evidence**

This study provides the first clear and direct comparison of f-IPV with full-dose IPV as primary immunisation of infants against poliovirus in delayed schedules. It confirms that immunogenicity can be conferred using f-IPV doses to make such dose-sparing immunisations affordable in low-income and middle-income countries provided the immunisation schedule is adapted to ensure maternal antibodies do not interfere with the response. As such, it will inform policy makers to formulate future immunisation recommendations following global eradication of wild-type polioviruses and cessation of all OPV use.

Several clinical trials have contributed data to the evidence base to support the adoption of combined bivalent OPV and IPV schedules.^7^, ^8^, ^9^, ^10^, ^11^ Most low-income countries now include one IPV dose with their bivalent OPV primary series. IPV introduction was delayed in approximately 40 low risk countries because of restricted global manufacturing capacity resulting in supply constraints. Faced with supply and cost constraints the WHO SAGE and regional Technical Advisory Groups recommended alternative IPV vaccination schemes be introduced, including intradermal application of fractional IPV (f-IPV) doses rather than full-dose IPV.^12^, ^13^ Although the supply situation has improved,^14^ the potential global withdrawal of all OPV following the declaration of global eradication of all wild-type polioviruses will necessitate increased use of IPV or f-IPV as the sole source of poliovirus immunity for infants.

Alfaro-Murillo and colleagues^15^ have suggested that OPV withdrawal could be done in regions, such as the Americas, where paralytic disease is entirely caused by vaccine-associated paralytic poliomyelitis and circulating vaccine-derived polioviruses. There is no risk of vaccine-associated paralytic poliomyelitis or emergence of vaccine-derived poliovirus associated with IPV use. Alfaro-Murillo and colleagues^15^ also acknowledged that a switch from OPV to IPV in some regions will be difficult to achieve because of cost and the shortfall in supply of IPV. This highlights the urgent need for scientific evidence to clarify the optimal IPV scheme to implement for the long term, following cessation of all OPV use. This study aimed to compare immune responses to intramuscular full-dose IPV with intradermal f-IPV in two different schedules in poliovirus-naive infants in Panama and the Dominican Republic where there have been no wild poliovirus or detectable circulating vaccine-derived polioviruses transmission since the 2000–01 outbreak.^16^ Infant vaccination schedules vary between the two countries. In 2014, Panama replaced OPV with hexavalent (diphtheria-tetanus-pertussis-hepatitis B-IPV-*Haemophilus influenzae* type b) combination vaccine for the three dose infant schedule, with bivalent OPV boosters at 18 months and 4 years of age. Whereas, the Dominican Republic uses IPV at 2 months followed by bivalent OPV at 4, 6, 18 months, and 4 years of age. Absence of any outbreak response use of poliovirus type 2 OPV in these two countries provided an ideal epidemiological setting to study poliovirus vaccine immunogenicity that simulates the post-OPV era.

## Methods

### Study design and participants

This randomised, open-label, multicentre, phase 3, non-inferiority trial was done at two urban sites in Panama and one urban site in the Dominican Republic. Eligible participants were healthy 6-week-old infants with no signs of febrile illness or known allergy to vaccine components. Infants were excluded if they had received previous poliovirus vaccination, had a known HIV infection, had any blood disorder contraindicating intramuscular or intradermal injections, or if any household contacts had been vaccinated with OPV in the previous 4 weeks.

Institutional Review Boards of each study centre and the respective national committees approved the study protocol. Parents or guardians of all participating infants provided signed informed consent before enrolment and the study was done according to International Council for Harmonisation and Good Clinical Practice guidelines. The trial is registered on ClinicalTrials.gov, NCT03239496.

### Randomisation and masking

At enrolment infants were randomly assigned (1:1:1:1, 1:1:1:2, or 2:1:1:1) in varying block sizes of four or five to one of four vaccination schedules: three IPV doses (three IPV group), two IPV doses (two IPV group), three f-IPV doses (three f-IPV group), or two f-IPV doses (two f-IPV group). Study nurses were trained in the injection technique for intradermal administration. Because of the nature of the trial assessing four different vaccination schedules, administrators, nurses giving the vaccine, and the infants and their parents or guardians were not masked to treatment allocation, but laboratory staff responsible for measuring the immunogenicity outcomes for the primary outcomes were masked to treatment group and timepoint the sample was taken.

### Procedures

**Figure 1:**
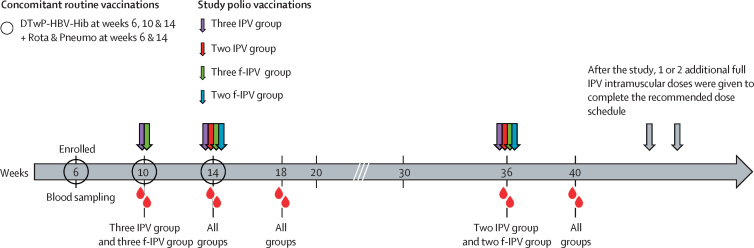
Study design The experimental study schedule used in the four study groups in both the Dominican Republic and Panama illustrating the administration of IPV and f-IPV, the concomitant routine vaccinations, and the blood sampling timepoints for assessment of poliovirus neutralising antibodies. IPV was administered intramuscularly. f-IPV was administered intradermally. f-IPV was one-fifth of a normal IPV dose. IPV=inactived poliovirus vaccine. f-IPV=fractional-inactived poliovirus vaccine. DTwP=diptheria-tetanus-whole-cell pertussis vaccine. HBV=hepatitis B vaccine. Hib=*Haemophilus influenzae* type b vaccine. Rota=rotavirus vaccine. Pneumo=pneumococcal vaccine.

The study vaccine was a WHO-prequalified Salk IPV vaccine (Poliomyelitis Vaccine [Inactivated], Serum Institute of India, Pune, India), which is representative of all available IPV vaccines. Each 0·5 mL dose contained 40 D antigen units Mahoney strain (poliovirus type 1), 8 D antigen units MEF-1 strain (poliovirus type 2), and 32 D antigen units Saukett strain (poliovirus type 3), 2·5 mg of 2-phenoxyethanol, and up to 12·5 μg of formaldehyde. Full intramuscular IPV doses were administered in the anterolateral aspect of the thigh using a standard needle and syringe. Fractional doses, one-fifth of the full dose presented in 0·1 mL, were administered intradermally in the upper arm using a 0·1 mL autodisable syringe. Vaccines were administered to the two f-IPV group on weeks 14 and 36; to the three f-IPV group on weeks 10, 14, and 36; to the two IPV group on weeks 14 and 36 weeks; and to the three IPV group on weeks 10, 14, and 36. For this study infants in both countries received the same routine vaccines in the same schedules (figure 1). This was necessary because the standard hexavalent IPV-containing combination vaccine had to be replaced with a pentavalent one (diphtheria, tetanus, whole-cell pertussis, hepatitis B, and *Haemophilus influenzae* type b) to allow the IPV and f-IPV vaccines to be administered separately. When necessary, the polio vaccines were administered concomitantly with routine infant vaccines (pentavalent combination, pneumococcal, rotavirus, and influenza), which were administered according to the national immunisation recommendations (figure 1). Infants in the two-dose groups (the two IPV group and the two f-IPV group) received additional IPV vaccinations after the study to ensure they met these requirements.

Infants were monitored for 30 min after vaccination, then parents recorded solicited local reactions (pain, induration, and redness) and body temperature for 7 days; unsolicited adverse events were monitored throughout the study in e-diaries. Parents were to immediately report the occurrence of any serious adverse events or important medical events throughout the duration of the study. Serious adverse events were defined as death or events that were either life-threatening, required hospitalisation, or resulted in persistent or significant disability or incapacity. Important medical events were medically significant events defined in the protocol (appendix 2) that did not meet any serious adverse events criteria but required medical consultation or intervention.

Four blood samples were taken from each participant: at 10 or 14 weeks of age before the first vaccination, and at 18, 36, and 40 weeks of age (figure 1). Sera were stored at temperatures of −20°C or lower for measurement of neutralising antibodies against polioviruses type 1, type 2, and type 3 using the WHO standard microneutralisation assay (WHO EPI GEN 93.9) adapted at the Centres for Disease Control and Prevention laboratories (Atlanta, GA, USA), as previously described.^17^ Seroprotection rates (group proportions with a reciprocal neutralising antibody titre ≥8 for each poliovirus type) and group geometric mean titres at each timepoint were calculated using a logarithmic (base 2) scale. Immune responses were expressed as seroconversion rates (total proportions of each group that changed from seronegative to seropositive, or baseline seropositive infants who displayed a four-times or higher increase in antibody titres after vaccination assuming an exponential decay of maternal antibodies with a half-life of 24 days).

### Outcomes

The primary objective of the study was to determine whether the immune response induced against poliovirus type 1 and type 2—the two poliovirus types that as wild polioviruses or circulating vaccine-derived poliovirus are responsible for all current cases of paralytic disease—4 weeks after completion of the primary series were similar between infants who received vaccine schedules that consisted of two or three doses of IPV, or two or three doses of f-IPV, to decrease the number of doses required to provide an adequate standard of immune protection in the community.

Three co-primary outcomes concerning poliovirus types 1 and 2 were to determine if seroconversion rates at 40 weeks of age after a two-dose regimen of intradermally administered f-IPV were non-inferior to a corresponding two-dose regimen of intramuscular IPV; if seroconversion rates at 40 weeks of age after a two-dose IPV regimen were non-inferior to those after a three-dose IPV regimen; and if seroconversion rates after a two-dose f-IPV regimen are non-inferior to those after a three-dose f-IPV regimen. Secondary outcomes included assessments of superiority of seroconversion rates for poliovirus types 1 and 2 of the four regimens with different administration schedules, immunogenicity of different regimens in terms of neutralising antibody titres, and safety. Exploratory outcomes included the primary and secondary outcomes for poliovirus type 1 and type 2, applied to poliovirus type 3.

### Statistical analysis

To maintain statistical power without increasing the required number of participants, primary objective comparisons were restricted to poliovirus type 1 and type 2 considering the current global epidemiology of polioviruses. They also represent examples of poliovirus types that are included (type 1) and excluded (type 2) from the bivalent OPV vaccines used in both study countries in case the ongoing use of these live viruses in the study environment influences the responses. Before the study, seroconversion rates of 80% for the three IPV group, 96% for the two IPV group, 64% for the three f-IPV group, and 95% for the two f-IPV group after the second dose were assumed, and seroconversion rates of 99% for the three IPV group and 96% for the three f-IPV group after the third dose were assumed. Sample sizes were chosen such that the primary non-inferiority comparison would have 80% or higher power for a joint comparison of poliovirus type 1 and type 2 (90% for each type individually). Secondary comparisons of superiority and non-inferiority also had 80% power or higher, except for the comparison between the two f-IPV group and the three IPV group, which could not have been meaningfully increased without an infeasibly large sample size. The sample size for each group was further increased, assuming a 10% dropout and non-evaluability rate. All non-inferiority comparisons of seroconversion rates between vaccine regimens and vaccine types were made with the lower bound of two-sided score-based CIs (α=0·05) with a 10% non-inferiority margin. The non-inferiority margin was chosen via the fixed-margin method, including preservation of 90% benefit (lower CI of seroconversion rate from previous studies),^18^, ^19^ expected for comparator groups involved in primary objective comparisons. All comparisons were made with the sample taken 4 weeks after the last vaccination for each specified regimen, including those in which the participant age differed at this time point (eg, the three IPV group *vs* the two IPV group after the first two doses), accounting for the decay of maternally derived antibody as previously described. Superiority comparisons of seroconversion rates were made using one-sided Fisher's exact test (α=0·025). Comparisons of neutralising antibody titres between regimens at the same timepoint were done using geometric mean titre ratios, facilitated by an analysis of covariance model of the log_2_ titre to adjust for the baseline concentration and study site as fixed effects. Non-inferiority of regimen 1 to regimen 2 was declared if the lower 95% bound of the two-sided CI for the geometric mean titre ratio is greater than 0·67, selected as the more stringent of common margins for non-inferiority evaluations of geometric mean titres between vaccines or vaccine regimens in accordance with WHO guidance on the clinical evaluation of vaccines. No adjustment for multiple comparisons was done in this study.

### Role of the funding source

ASB and JM were full-time employees of the Bill & Melinda Gates foundation, which provided grant funding for the study. RR was a full-time employee of Fighting Infectious Diseases in Emerging Countries, the study sponsor. All were responsible for the study design and protocol. Contract research organisations monitored the trial and managed the data (VaxTrials) and did the statistical analysis (Assign DMB). All authors had full access to the data, prepared the manuscript (with the assistance of an independent professional medical writer funded by the study sponsor), and agreed to its submission.

## Results

**Figure 2:**
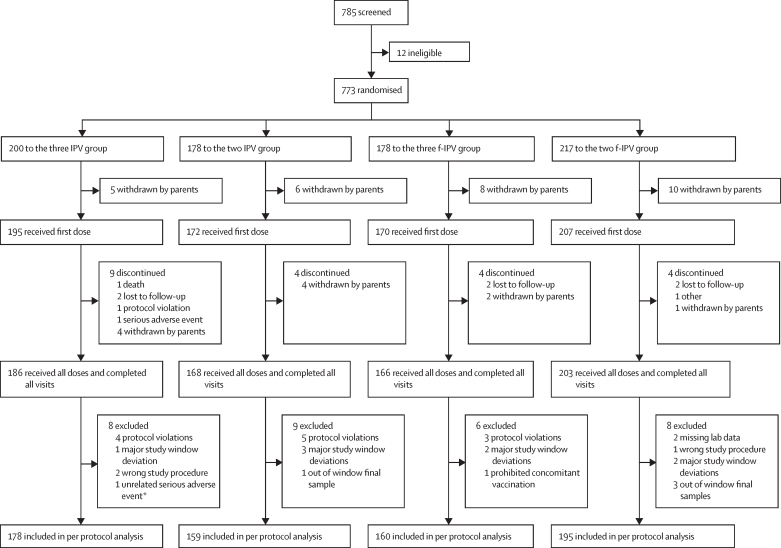
Trial profile IPV=inactived poliovirus vaccine. f-IPV=fractional-inactived poliovirus vaccine. *Infant had sepsis and fully recovered. Sepsis occurred at 18 weeks and could have interfered with the immune response to vaccination. IPV was administered intramuscularly. f-IPV was administered intradermally.

From Oct 23, 2017, to Nov 13, 2018, we enrolled 773 (372 [48%] girls) infants in Panama and the Dominican Republic. Enrolment was balanced by site in Panama and the Dominican Republic and infants were randomly assigned to the four study groups (two f-IPV group n=207, three f-IPV group n=170, two IPV group n=172, and three IPV group n=195). 744 (96%) received at least one polio vaccination (367 IPV and 377 f-IPV) representing the safety population (figure 2). Of whom, 723 (94%) completed their vaccination regimens at week 40, and 692 (90%) were eligible for the immunogenicity analysis (figure 2). Baseline characteristics and demographics in the safety population are reported in table 1. Seroconversion rates by poliovirus type for each vaccine schedule are reported in table 2. In a combined assessment of the three IPV group and the three f-IPV group at 10 weeks of age, 163 (45%) of 365 infants were seropositive for poliovirus type 1, 168 (46%) seropositive for poliovirus type 2, and 75 (21%) seropositive for poliovirus type 3. In a combined assessment of antibodies in the two IPV group and the two f-IPV group, the baseline seropositivity rates at 14 weeks of age were 98 (26%) of 379 infants for poliovirus type 1, 92 (24%) for poliovirus type 2, and 41 (11%) for poliovirus type 3, illustrating the waning of maternal antibodies when compared with the combined three dose groups at 10 weeks.

**Table 1:** Baseline characteristics of all patients that received at least one vaccine

		**Three IPV group (n=195)**	**Two IPV group (n=172)**	**Three f-IPV group (n=170)**	**Two f-IPV group (n=207)**
Mean age, weeks (SD)		5·5 (0·70)	5·5 (1·02)	5·5 (0·68)	5·4 (1·05)
Boys		104 (53%)	83 (48%)	93 (55%)	105 (51%)
Girls		91 (47%)	89 (52%)	77 (45%)	102 (49%)
Ethnicity					
	Black	3 (2)	1 (1)	7 (4)	3 (1)
	Hispanic	105 (54)	107 (62)	100 (59)	131 (63)
	Latin American	84 (43)	64 (37)	62 (36)	69 (33)
	White	3 (2)	0 (0)	1 (1)	4 (2)
Mean weight, kg (SD)		4·4 (0·63)	4·4 (0·63)	4·4 (0·66)	4·4 (0·55)
	Range, kg	3·2–6·3	2·9–6·5	2·2–6·0	2·0–5·9
Baseline seropositivity rate*					
	Poliovirus type 1	84 (43%)	46 (27%)	79 (46%)	52 (25%)
	Poliovirus type 2	97 (50%)	43 (25%)	71 (42%)	49 (24%)
	Poliovirus type 3	44 (23%)	17 (10%)	31 (18%)	24 (12%)

Data are n (%) unless otherwise specified. The three IPV group received intramuscular vaccine at 10, 14, and 36 weeks. The two IPV group received intramuscular vaccine at 14 and 36 weeks. The three f-IPV group received intradermal vaccine at 10, 14, and 36 weeks. The two f-IPV group received intradermal vaccine at 14 and 36 weeks. f-IPV was one-fifth of a normal IPV dose. IPV=inactivated poliovirus vaccine. f-IPV=fractional inactivated poliovirus vaccine.

* Baseline measured at 10 weeks for the three dose regimens and at 14 weeks for the two dose regimens.

**Table 2:** Seroconversion rates by poliovirus type in the per-protocol population of each vaccine schedule group

	**Poliovirus type 1n/N (% [95% CI])**	**Poliovirus type 2 n/N (% [95% CI])**	**Poliovirus type 3 n/N (% [95% CI])**
Three IPV group at week 18 after two doses	172/180 (96% [91·4–98·1])	160/180 (88% [83·4–93·1])	169/18093·9 (89·3–96·9)
Three IPV group at week 40 after three doses	178/178 (100% [97·9–100])	178/178 (100% [97·9–100])	178/178100 (97·9–100)
Two IPV group at week 40 after two doses	152/154 (99% [95·4–99·8])	153/154 (99% [96·4–100])	150/154 (97% [93·5–99·3])
Three f-IPV group at week 18 after two doses	134/161 (83% [76·5–88·6])	135/161 (84% [77·2–89·2])	118/161 (73% [65·8–79·9])
Three f-IPV group at week 40 after three doses	158/160 (99% [95·6–99·8])	160/160 (100% [97·7–100])	150/160 (94% [88·8–97·0])
Two f-IPV group at week 40 after two doses	186/194 (96% [92·0–98·2])	190/194 (98% [94·8–99·4])	164/194 (85% [78·7–89·3])

Data are n (%) unless otherwise specified. The three IPV group received intramuscular vaccine at 10, 14, and 36 weeks. The two IPV group received intramuscular vaccine at 14 and 36 weeks. The three f-IPV group received intradermal vaccine at 10, 14, and 36 weeks. The two f-IPV group received intradermal vaccine at 14 and 36 weeks. f-IPV was one-fifth of a normal IPV dose. IPV=inactivated poliovirus vaccine. f-IPV=fractional inactivated poliovirus vaccine.

The three co-primary immunogenicity outcomes regarding seroconversion rates for poliovirus types 1 and 2 were positively met (table 3)—namely, non-inferiority of the two f-IPV group compared with the two IPV group, the two IPV group compared with the three IPV group, and the two f-IPV group compared with the three f-IPV group. In all cases the lower confidence bound of the absolute differences in the percentages of immune responses was higher than −10%. Exploratory non-inferiority comparisons showed that three doses of f-IPV (the three f-IPV group) were non-inferior to two (the two IPV group) or three (three IPV group) doses of IPV for the type 1 and type 2 seroconversion rates (table 3).

**Table 3:** Superiority and non-inferiority comparisons between the per-protocol population of each vaccine schedule group

	**Poliovirus type 1 (95% CI)**	**Poliovirus type 2 (95% CI)**	**Poliovirus type 3 (95% CI)**
**Non-inferiority comparisons**			
Two f-IPV group *vs* two IPV group	−2·2 (−6·3 to 1·8)	−0·8 (−4·1 to 2·6)	−12·2 (−18·4 to −6·5)
Two IPV group *vs* three IPV group	−1·9 (−5·4 to 0·3)	−1·3 (−4·5 to 0·9)	−3·1 (−7·2 to −1·0)
Two f-IPV group *vs* three f-IPV group	−2·9 (−6·8 to 0·8)	−2·1 (−5·2 to 0·3)	−9·1 (−15·7 to −2·7)
Three f-IPV group *vs* three IPV group	−1·3 (−4·4 to 0·9)	0 (−2·9 to 2·5)	−6·3 (−11·1 to −3·4)
Two f-IPV group *vs* three IPV group	−4·1 (−7·9 to −1·9)	−2·1 (−5·2 to 0·1)	−15·4 (−21·1 to −11·0)
Three f-IPV group *vs* two IPV group	0·6 (−2·8 to 4·3)	1·3 (−1·1 to 4·5)	−3·1 (−8·4 to 1·7)
**Superiority comparisons**			
Two IPV group *vs* three IPV group after two doses	2·6 (−1·5 to 6·9)	9·9 (5·2 to 15·5)†	3·0 (−1·8 to 7·9)
Two f-IPV group *vs* three f-IPV group after two doses	12·7 (6·6 to 19·6)†	14·1 (8·6 to 20·8)†	11·3 (2·9 to 20·0)‡

The three IPV group received intramuscular vaccine at 10, 14, and 36 weeks. The two IPV group received intramuscular vaccine at 14 and 36 weeks. The three f-IPV group received intradermal vaccine at 10, 14, and 36 weeks. The two f-IPV group received intradermal vaccine at 14 and 36 weeks.

*Using values at 18 weeks for the three dose regimens, 4 weeks after administration of second dose.

† p<0·0001.

‡ p=0·0062.

When poliovirus type 3 responses were analysed, non-inferiority was only shown between the two IPV group and the three IPV group (table 3). In most comparisons between the f-IPV and IPV regimens, the f-IPV type 3 responses were inferior, with the exception of the three f-IPV group compared with the two IPV group.

In superiority analyses done to assess the influence of timing of vaccinations, two IPV doses at 14 and 36 weeks elicited significantly higher seroconversion rates against type 2 poliovirus than when administered at 10 and 14 weeks, but responses to poliovirus type 1 and type 3 did not differ between these two-dose schedules. Seroconversion rates to all poliovirus types were all significantly higher when two f-IPV doses were administered at 14 and 36 weeks rather than 10 and 14 weeks (table 3).

**Figure 3:**
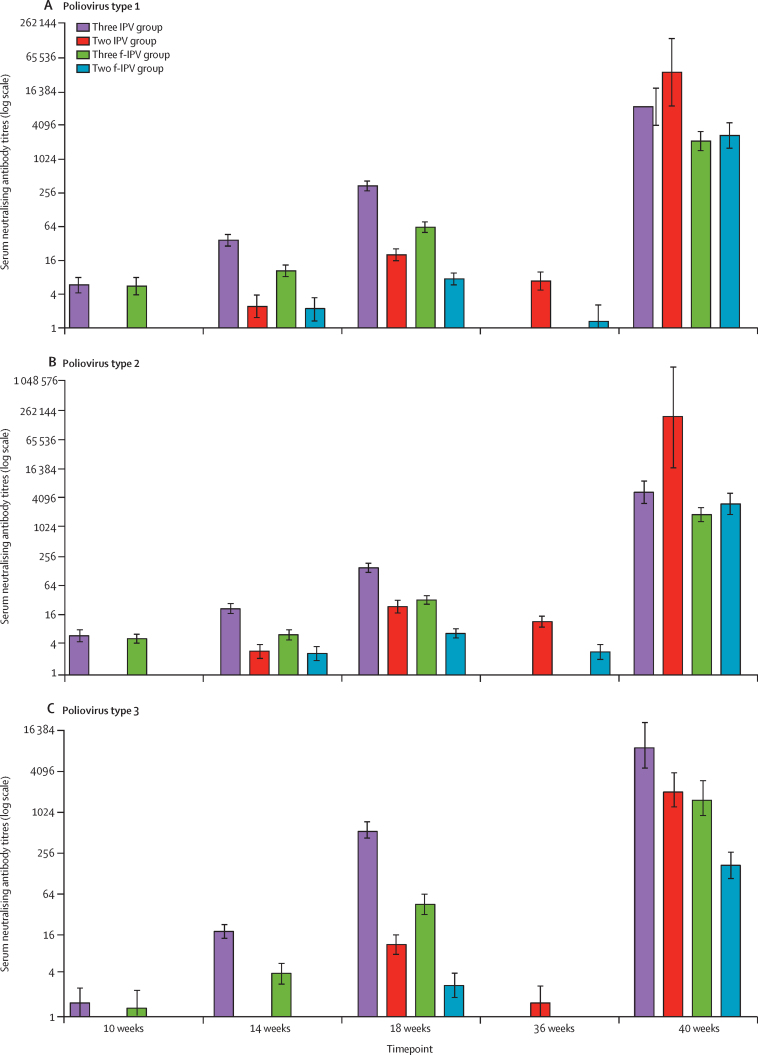
Geometric mean titres of poliovirus type-specific serum neutralising antibodies in each group at each sampling timepoint Error bars are 95% CIs. f-IPV was one-fifth of a normal IPV dose. IPV was administered intramuscularly. f-IPV was administered intradermally. IPV=inactived poliovirus vaccine. f-IPV=fractional-inactived poliovirus vaccine.

Following vaccination in the three IPV group and the three f-IPV group, antibody geometric mean titres increased for all three poliovirus types from baseline; antibodies at baseline were presumed to be maternal. In these groups geometric mean titres progressively increased at 14 and 18 weeks after the first and second vaccinations, and peaked at 40 weeks after the third dose at 36 weeks (figure 3). In the two IPV group and the two f-IPV group baseline was at 14 weeks, when maternal antibody titres were lower than in the three IPV group and the three f-IPV group. In three dose regimens, IPV vaccination induced higher titres than f-IPV at 18 weeks, 4 weeks after vaccination. Titres then waned by week 36, when the second dose increased poliovirus type 1 and 2 antibodies such that the three f-IPV group and the two f-IPV group geometric mean titres were similar at 40 weeks. The highest geometric mean titres to poliovirus type 1 and type 2 were reported in the two IPV group after two IPV doses (at 40 weeks), but the highest geometric mean titre to type 3 was reported in the three IPV group at 40 weeks.

No reactions within 30 min of vaccination occurred. One infant in the Dominican Republic in the three IPV group died because of shock and cardiorespiratory arrest 15 weeks after the second IPV vaccination, but this was not considered to be causally associated with the study. There were 38 serious adverse events (ten in the Dominican Republic and 28 in Panama) reported in 32 infants, 19 events in 16 infants in both the two IPV groups and the two f-IPV groups (table 4). One infant with three serious adverse events (anaemia, diarrhoea, and sepsis) due to the congenital condition adrenal hyperplasia received one vaccination and was then withdrawn, but they remained in otherwise good health at final contact. None of the serious adverse events or 14 important medical events (eight mild and six moderate) reported in eight infants were considered to be causally associated with vaccination.

**Table 4:** Summary of adverse events and important medical events

			**Three IPV group (n=195)**	**Two IPV group (n=172)**	**Three f-IPV group (n=170)**	**Two f-IPV group (n=207)**
Patients with serious adverse events (%; number of events)*			10 (5%; 12 events)	6 (3%; 7 events)	7 (4%; 10 events)	9 (4%; 9 events)
Deaths			1 (1%)	0	0	0
Life threatening adverse events			0	0	0	1 (1%)
Required hospitalisation			8 (4%)	5 (3%)	7 (4%)	9 (4%)
Congenital anomaly or birth defect			1 (1%)	1 (1%)	0	0
Patients with important medical events (%; number of events)*			4 (2%; 8 events)	0	2 (1%; 4 events)	2 (1%; 2 events)
Fever†			0	1 (1%)	1 (1%)	1 (1%)
Local adverse events						
	Total number of doses		565	338	499	410
		Pain	1 (0·2%)	1 (0·3%)	12 (2·4%)	1 (0·2%)
		Redness	5 (0·9%)	2 (0·6%)	10 (2·0%)	9 (2·2%)
		Inflammation	0	1 (0·3%)	2 (0·4%)	1 (0·2%)

Data are n (%). The three IPV group received intramuscular vaccine at 10, 14, and 36 weeks. The two IPV group received intramuscular vaccine at 14 and 36 weeks. The three f-IPV group received intradermal vaccine at 10, 14, and 36 weeks. The two f-IPV group received intradermal vaccine at 14 and 36 weeks.

* All events were unrelated to study regimens.

† All cases of fever were reported after the first vaccination.

Solicited injection site reactions were infrequent in all groups (table 4); in the 739 infants monitored after their first vaccination there were six reports of tenderness, ten of redness, and four of inflammation, all mild to moderate and transient. Mild injection site redness was reported in nine of 724 infants after the second vaccination and seven of 349 after their third vaccination, with no reports of pain or inflammation in these groups. There were three reports of mild fever—one each in the two IPV, the three f-IPV, and the two f-IPV groups—after the first vaccination but none after subsequent doses.

## Discussion

Following certification of the global eradication of wild poliovirus type 1 and interruption of circulating vaccine-derived poliovirus transmission, it will be crucial to maintain immunity against poliovirus for the immediate period after eradication. Our study provides novel data that can inform global, regional, and national policy decisions on IPV-only schedules for the post-eradication era, with an option for dose-sparing and cost-saving in settings where routine use of full doses of IPV are challenging because of cost or availability. We show that two intradermal f-IPV doses can elicit robust immune responses—as shown by the seroconversion rate for poliovirus type 1 and type 2—that are non-inferior to two full doses of IPV, but these responses are dependent on the age of the infant at the time of administration and also probably affected by the time interval between vaccinations. Seroconversion rates to two doses of f-IPV given at 14 and 36 weeks were significantly superior for all three virus types than when the doses were given at 10 and 14 weeks. When given at 14 and 36 weeks, f-IPV was non-inferior to IPV for poliovirus type 1 and type 2, although rates for type 3 were inferior, and geometric mean titres were lower. These observations of equivalent seroconversion rates are important because seroconversion to polioviruses at any timepoint confers protection against paralytic disease.^20^, ^21^

When IPV is administered alone or in a combination with diphtheria-tetanus-pertussis-based vaccines it has proven to be safe, with no causal association with any adverse events other than temporary, minor, local reactions, such as erythema (<1%), induration (3–11%), or tenderness (14–29%).^22^, ^23^, ^24^ Our data confirm these observations, with no vaccine-related serious adverse event or important medical event. Reactogenicity mainly consisted of infrequent, mild-to-moderate injection site reactions, with only three reports of mild fever, all after the first vaccination.

With global eradication of wild polioviruses, all live polio vaccines will be withdrawn from routine use to eliminate the risk of emergence of vaccine-derived polioviruses and vaccine-associated paralytic poliomyelitis. Countries using bivalent OPV will switch to IPV-only schedules for routine infant immunisations. Faced with the challenges of increasing manufacturing capacity to meet future global requirements for IPV^13^ and the higher cost of IPV per dose than OPV, the use of intradermal f-IPV is an attractive option.^22^ IPVs manufactured from wild Salk poliovirus strains or attenuated Sabin poliovirus strains inactivated with formaldehyde are available and are generally considered to be equivalent to each other in terms of immunogenicity. These inactivated vaccines can be administered by subcutaneous or intramuscular injection, often in diphtheria-tetanus-pertussis-based combinations, and fractional doses of stand-alone IPV can be administered intradermally. However, all IPV vaccines have been licensed based on three-dose infant schedules with the first dose administered in the second month of life and there is only a small amount of information on the optimal schedule for f-IPV.

Studies with alternative f-IPV schedules have generally shown lower seroconversion rates than full IPV doses depending on age at administration, but two f-IPV doses can substitute for one full IPV dose.^24^, ^25^ Studies in Bangladesh^26^ and Cuba^27^ found intradermal f-IPV elicited significantly lower seroconversion rates and geometric mean titres than intramuscular IPV for all three poliovirus types when administered at 6, 10, and 14 weeks of age. In another study in Bangladesh, a priming dose of f-IPV 6 weeks before subsequent OPV was also inferior to an IPV schedule.^28^ Another Cuban study found that one or two doses of f-IPV at 4 and 8 months induced significantly lower seroconversion rates than IPV, but there was evidence of immune priming with initial doses of f-IPV.^19^ The same investigators showed that responses to intramuscular f-IPV at 4 and 8 months were non-inferior to the intradermal route.^29^ A three-dose primary series of intradermal f-IPV in infants at 2, 4, and 6 months of age elicited similar seroconversion rates for each of the three poliovirus types as full dose IPV, but significantly lower titres.^18^

Intradermal vaccine administration will require training of health-care staff, but there are examples to show that this was done successfully with the introduction of intradermal f-IPV.^30^ Our study provides novel clinical data on the administration of two or three doses of IPV or f-IPV in delayed schedules proposed by SAGE. We found that two doses of intramuscular IPV or intradermal f-IPV at 14 and 36 weeks provide acceptable seroconversion rates against all three poliovirus types although geometric mean titres—most notably to poliovirus type 3—were lower. However, a three-dose f-IPV regimen might be considered ideal given the suboptimal immunogenicity against poliovirus type 3 and lower geometric mean titres overall with two f-IPV doses. Consistent with SAGE recommendations for future two-dose regimens, our study supports delaying the first IPV dose until 14 weeks of age to minimise interference by maternally derived antibodies.

The study was open-label because of the evident differences in presentation and mode of administration of IPV and f-IPV, but the laboratory personnel responsible for measuring immunogenicity for the primary objective were masked to treatment group and timepoints. Other assessments, including local reactions arising from the intradermal administration of f-IPV compared with the intramuscular administration of IPV, might have been affected. For example, more frequent observation of severe redness or induration at the injection site might arise from greater overall reporting of such local reactogenicity because of the different route of administration.

Our study is the first to inform decisions on polio immunisation schedules for an era with no elective use of OPVs. The findings offer strong evidence of the potential to mitigate cost and supply constraints related to IPV for the concluding phase of the eradication programme. We report that near-universal immune responses can be elicited with two full doses of intramuscular IPV when given in the delayed schedules reported in this study, implying substantial cost and supply savings because the current practice is to use four or more full doses of IPV in routine immunisation schedules in countries where OPV is not being used. We also report that two f-IPV doses administered via the intradermal route are protective against the two serotypes that are circulating as wild-type or circulating vaccine-derived poliovirus, and that three f-IPV doses are protective against all three polio serotypes, which will provide additional cost and supply savings. Given the rapidly evolving landscape of poliovirus vaccine development, with changing epidemiology of vaccine-derived poliovirus following global type 2 OPV withdrawal in 2016 and ongoing wild poliovirus type 1 circulation, policy decisions on novel IPV-only schedules will have to be optimised as data on newer IPV formulations and delivery methods, including the option to deliver fractional doses via the intramuscular route, become available.^30^, ^31^

## Data sharing

Following complete publication, the data generated in this study will be made available to researchers through the Gates Open Research portal. For details see https://gatesopenresearch.org/for-authors/data-guidelines.

## Supplementary Material

Spanish translation of the abstract
