# Supplementary material for: Safety and immunogenicity of inactivated poliovirus vaccine schedules for the post-eradication era: a randomised open-label, multicentre, phase 3, non-inferiority trial
Source: Lancet Infect Dis. 2021 Apr;21(4):559–68. doi: 10.1016/S1473-3099(20)30555-7 (PMC7992032; doi:10.1016/S1473-3099(20)30555-7)
Supplement: Spanish translation of the abstract [file mmc1.pdf]

THE LANCET
Infectious Diseases

Supplementary appendix 1

This translation in Spanish was submitted by the authors and we reproduce it as supplied. It has not been peer reviewed. *The Lancet*'s editorial processes have only been applied to the original in English, which should serve as reference for this manuscript.

Supplement to: Bandyopadhyay AS, Gast C, Rivera L, et al. Safety and immunogenicity of inactivated poliovirus vaccine schedules for the post-eradication era: a randomised open-label, multicentre, phase 3, non-inferiority trial. *Lancet Infect Dis* 2020; published online Oct 23. https://doi.org/10.1016/S1473-3099(20)30555-7.

Los autores nos proporcionaron esta traducción al español y la reproducimos tal como nos fue entregada. No la hemos revisado. Los procesos editoriales de *The Lancet* se han aplicado únicamente al original en inglés, que debe servir de referencia para este manuscrito.

Resumen: En seguimiento del Plan Global de Erradicación de los poliovirus salvajes, los países que utilizan las vacunas de polio oral deben cambiar a vacuna de polio inactivada (IPV) o dosis fraccionada de IPV (f-IPV; una f-IPV representa un quinto de la dosis normal de IPV), pero la provisión de la IPV y su costo requieren estrategias de ahorro. Se compararon los esquemas de inmunización de f-IPV e IPV para generar información sobre la mejor estrategia en la era post erradicación.

Métodos: este estudio randomizado, abierto, multicéntrico, de fase 3 y de no inferioridad, se realizó en 2 centros en Panamá y uno en República Dominicana. Los participantes elegidos fueron lactantes de 6 semanas de edad, sin fiebre o alergias conocidas a algún componente de las vacunas. Los lactantes fueron randomizados utilizando un sistema computarizado en bloques (1:1:1:1, 1:1:1:2, 2:1:1:1) que lo asignó a 1 de 4 grupos hasta que estuvo completo para recibir: dos dosis de f-IPV intradérmica (Grupo 2 dosis de f-IPV); o tres dosis de f-IPV intradérmica (Grupo 3 dosis de f-IPV); o dos dosis de IPV intramuscular (Grupo 2 dosis de IPV); o tres dosis de IPV intramuscular (Grupo 3 dosis de IPV). La evaluación primaria en la población por protocolo fue la tasa de seroconversion de los anticuerpos neutralizantes para poliovirus tipo 1 y tipo 2 basales y a las 40 semanas (4 semanas después de la segunda o tercera dosis de vacuna) para permitir el criterio de comparación de no-inferioridad y eventualmente superioridad entre los regímenes y las vacunas. Las tres comparaciones co-primarias sobre los poliovirus tipo 1 y 2 que se realizaron fueron: si la tasa de seroconversión a las 40 semanas de edad luego del esquema de dos dosis (administradas a la semana 14 y 36) de la f-IPV intradérmica fue no-inferior a la correspondiente de dos dosis con la administración de IPV intramuscular; si la tasa de seroconversión a las 40 semanas de edad luego del esquema de dos dosis de IPV (semana 14 y 36) fue no-inferior a la de aquellos que recibieron 3 dosis de IPV (semana 10, 14 y 36); y la tasa de seroconversión luego de dos dosis de f-IPV (semana 14 y 36) fue no-inferior a aquellos luego del esquema de tres dosis de f- IPV (semanas 10, 14 y 36). El margen de no-inferioridad se definió como -10% para el nivel inferior de las dos colas del intervalo de confianza 95% de la diferencia en la tasa de seroconversión. Para la seguridad se evaluaron los eventos adversos serios y los eventos médicos importantes. Este estudio se registró en ClinicalTrials.gov, NCT03239496.

Resultados: del 23 de octubre de 2017 al 13 de noviembre de 2018, se enrolaron 773 lactantes (372 [48%] niñas) en Panamá y República Dominicana.

(Grupo 2 dosis de f-IPV n= 217, Grupo 3 dosis de f-IPV n=178, Grupo 2 dosis de IPV n=178 y Grupo 3 dosis de IPV n=200. 686 lactantes recibieron todas las dosis y esquemas y fueron incluidos en el análisis por protocolo. Se documentó no inferioridad para la tasa de seroconversión de poliovirus tipo 1 a la semana 40 para el esquema de 2 dosis de f-IPV (95·9% [95% CI 92·0–98·2]) versus el esquema de 2 dosis de IPV (98·7% [95·4–99·8]) y para el de tres dosis de f-IPV (98·8% [95·6–99·8]) versus el de 3 dosis de IPV (100% [97·9–100]). También la no inferioridad para la tasa de seroconversión de poliovirus tipo 2 a la semana 40 para el esquema de 2 dosis de f-IPV (97·9% [95% CI 94·8–99·4]) versus el esquema de dos dosis de IPV (99·4% [96·4–100]) y para el de tres dosis de f-IPV (98·8% [95·6–99·8]) versus el de 3 dosis de IPV (100% [97·9–100]). Las tasas de seroconversión del esquema de 2 dosis de f-IPV fueron superiores estadísticamente para poliovirus 1 cuatro semanas después de la última dosis en el esquema de 14 y 36 semanas (95·9% [92·0–98·2]) comparado con el de 10 y 14 semanas (83·2% [76·5–88·6]; p=0·0062). Esta diferencia estadística también se documentó para el esquema de 14 y 36 semanas para poliovirus tipo 2 (14 y 36 semanas 97·9% [94·8–99·4] vs 10 y 14 semanas 83·9% [77·2–89·2]; p=0·0062) y para poliovirus tipo 3 (14 y 36 semanas 84·5% [78·7–89·3] vs 10 y 14 semanas 73·3% [65·8–79·9]; p=0·0062). En el grupo de IPV, el esquema de dos dosis administradas a la 14 y 36 semanas (99·4% [96·4–100]) fue superior al de 10 y 14 semanas (88·9% [83·4–93·1]; p<0·0001) para poliovirus tipo 2, pero no para el tipo 1 (14 y 36 semanas 98·7% [95·4–99·8] vs 10 y 14 semanas 95·6% [91·4–98·1]), o tipo 3 (14 y 36 semanas 97·4% [93·5–99·3] vs 10 y 14 semanas 93·9% [89·3–96·9]). No hubo eventos adversos serios o eventos médicos importantes relacionados reportados en ninguno de los grupos demostrando que la vía de administración no afecta la seguridad.

Interpretación: Nuestra observación sugiere que dos dosis de IPV o fIPV administradas a las 14 y 36 semanas de edad generan inmunidad adecuada contra los poliovirus tipo 1 y tipo 2 y ofrece amplia inmunidad y ahorro con el esquema de tres dosis de f-IPV. Estos nuevos datos clínicos generan información para las políticas globales de inmunización anti-poliomielíticas en la era post-erradicación.

Financiamiento: de la Fundación Bill y Melinda Gates.
