# Supplementary material for: Safety and immunogenicity of inactivated poliovirus vaccine schedules for the post-eradication era: a randomised open-label, multicentre, phase 3, non-inferiority trial
Source: Lancet Infect Dis. 2021 Apr;21(4):559–68. doi: 10.1016/S1473-3099(20)30555-7 (PMC7992032; doi:10.1016/S1473-3099(20)30555-7)
Supplement: Supplementary appendix 2 [file mmc2.pdf]

THE LANCET
Infectious Diseases

Supplementary appendix 2

This appendix formed part of the original submission and has been peer reviewed. We post it as supplied by the authors.

Supplement to: Bandyopadhyay AS, Gast C, Rivera L, et al. Safety and immunogenicity of inactivated poliovirus vaccine schedules for the post-eradication era: a randomised open-label, multicentre, phase 3, non-inferiority trial. *Lancet Infect Dis* 2020; published online Oct 23. https://doi.org/10.1016/S1473-3099(20)30555-7.

Final Version 1.0. Clinical Study Protocol

09-Aug- 2017

**A Phase 3, Open-label, Multicenter Randomized Trial to Evaluate Humoral Immunogenicity of Various Schedules of Intramuscular Full Dose and Intradermal Fractional Dose of Inactivated Polio Vaccine in Latin American Infants**

**Product**	IPV and f-IPV
**Protocol Number**	IPV-004 ABMG
**ClinicalTrials.gov Number**	NCT03239496
**Clinical Phase**	Phase 3
**Clinical Indication**	Injectable Polio Vaccine immunization
**Version**	1.0
**Issue Date**	09-Aug-2017

**Sponsor**	FIDEC – Fighting Infectious Diseases in Emerging Countries<br>2050 Coral Way, Suite 407<br>Miami, FL 33145 USA
**Sponsor Representative**	Ricardo Rüttimann, MD<br>Tel + 1 305 854 0075, Fax + 1 305 856 7847

SIGNATURES

Signature of Sponsor Representative

Title: **A Phase 3, Open-label, Multicenter Randomized Trial to Evaluate Humoral Immunogenicity of Various Schedules of Intramuscular Full-Dose and Intradermal Fractional Dose of Inactivated Polio Vaccine in Latin American Infants**

Name: Ricardo Rüttimann, MD
Tel + 1 305 854 0075, Fax + 1 305 856 7847

This Clinical Study Protocol and Amendments (if any) have been reviewed and approved by the Sponsor in order to ensure compliance with Good Clinical Practice.

Signature:

Date:

Signature of Statistician

Title: A Phase 3, Open-label, Multicenter Randomized Trial to Evaluate Humoral Immunogenicity of Various Schedules of Intramuscular Full Dose and Intradermal Fractional Doses of Inactivated Polio Vaccine in Latin American Infants

Name:	Chris Gast
Affiliation:	Gast Statistical Consulting, LLC
Address:	Woodinville, WA, USA

This Clinical Study Protocol and Amendments (if any) have been reviewed and approved by the study statistician in order to ensure that the protocol and any amendments cover all relevant statistical matters clearly and accurately, using technical terminology as appropriate.

Signature:

Date:

Signature of Investigator

Title: **A Phase 3, Open-label, Multicenter Randomized Trial to Evaluate Humoral Immunogenicity of Various Schedules of Intramuscular Full Dose and Intradermal Fractional Doses of Inactivated Polio Vaccine in Latin American Infants**

Name: Luis Rivera, MD

Affiliation: Hospital Maternidad Nuestra Señora de la Altagracia

Address: Calle Pedro Henríquez Ureña #49,
Santo Domingo, Dominican Republic

I have read this Clinical Study Protocol and Amendments (if any) and agree that it contains all information necessary for the proper conduct of the study. I will carry out the study as outlined herein, will complete the study within the designated time and in accordance with Good Clinical Practice.

Signature:

Date:

Signature of Investigator

Title: **A Phase 3, Open-label, Multicenter Randomized Trial to Evaluate Humoral Immunogenicity of Various Schedules of Intramuscular Full Dose and Intradermal Fractional Doses of Inactivated Polio Vaccine in Latin American Infants**

Name: Xavier Sáez-Llorens, MD

Affiliation: Cevaxin,

Address: Panama City and Chiriquí, Republic of Panama

I have read this Clinical Study Protocol and Amendments (if any) and agree that it contains all information necessary for the proper conduct of the study. I will carry out the study as outlined herein, will complete the study within the designated time and in accordance with Good Clinical Practice.

Signature:

Date:

PROTOCOL HISTORY

Protocol History<br>FIDEC			
**Document**	**Issue Date**	**Amendment Type**	**Comments**
Final version 1.0	09-August-2017	-NA	
		-	
		-	

TABLE OF CONTENTS

Title Page ... **1**
Signatures ... **2**
Protocol History ... **6**
Table of Contents ... **7**
Protocol Synopsis ... **10**
List of Abbreviations and Definitions of Terms ... **15**
Study Administrative Structure ... **17**
1. Introduction ... **18**
1.1 Background Information ... 18
1.2 Risk Benefit Analysis ... 19
1.2.1 Potential Risks ... 19
1.2.2 Potential Benefits ... 20
2. Study Objectives ... **21**
2.1 Primary Objectives ... 21
2.2 Secondary objectives ... 21
2.3 Exploratory Objectives ... 22
3. Study Endpoints ... **23**
3.1 Primary Endpoints ... 23
3.2 Secondary Endpoints ... 23
3.3 Exploratory Endpoints ... 23
4. Study Design ... **25**
4.1 Overview of Study Design ... 25
4.2 Discussion of Study Design ... 25
5. Selection of Study Population ... **26**
5.1 Inclusion Criteria ... 26
5.2 Exclusion Criteria ... 26
5.3 Criteria for Elimination from the Per-Protocol Population ... 26
5.4 Contraindications to Further Vaccination ... 26
6. Vaccines ... **28**
6.1 Physical Description of the Study Vaccine ... 28
6.2 Dose and Administration ... 28
6.3 Other Medication Administered in the Study ... 28
6.4 Packaging and Labeling ... 28
6.5 Storage and Vaccine Accountability ... 29
6.6 Randomization ... 29
6.7 Compliance ... 29
7. Prior and Concomitant Therapy ... **31**
8. Assessments ... **32**
8.1 Timing of Assessments ... 32
8.1.1 Collection and handling of blood samples ... 33
8.2 Immunogenicity ... 33

8.2.1 Immunogenicity Variables 33
8.3 Safety Evaluations 34
8.3.1 Serious Adverse Events 34
8.3.2 Important medical events (IMEs) 34
9. Study Termination/Completion 36
9.1 Study Completion 36
9.2 Removal of Subjects From Study or Investigational Product 36
9.2.1 Removal from Study 36
10. Statistical Methods 38
10.1 Statistical Analysis 38
10.1.1 Study Populations 38
10.1.2 Initial Characteristics of the Subject Sample 38
10.1.3 Immunogenicity Data 38
10.1.4 Safety Data 39
10.1.5 Missing Data 39
10.2 Determination of Sample Size 39
10.3 Immunogenicity Analysis 41
11. Serious Adverse Event Reporting 42
11.1 Definitions 42
11.1.1 Serious Adverse Events 42
11.1.2 Important Medical Event 42
11.2 Causality Assessment 43
11.3 Action Taken Regarding the Study Vaccine 44
11.4 Outcome 44
11.5 Recording of serious adverse events 44
11.6 Reporting of serious adverse events and important medical events to the sponsor 44
11.7 Reporting of Serious Adverse Events to Competent Authority/Ethics Committees 45
12. Ethical Aspects 46
12.1 Study-Specific Design Considerations 46
12.2 Regulatory Ethics Compliance 46
12.2.1 Investigator Responsibilities 46
12.2.2 Independent Ethics Committee or Institutional Review Board (IEC/IRB) 46
12.2.3 Informed Consent 48
12.2.4 Privacy of Personal Data 49
13. Administrative Requirements 50
13.1 Protocol Amendments/Notifications 50
13.2 Subject Identification and Enrollment Logs 50
13.3 Source Documentation 50
13.4 Case Report Form Completion 51
13.5 Monitoring 51
13.6 Data Management 51
13.7 Data Quality Assurance 52

13.8 On-Site Audits 52
13.9 Study Termination 53
13.10 Record Retention 53
13.11 Use of Information and Publication 53
13.12 Registration of Clinical Studies and Disclosure of Results 54
13.13 Investigator Indemnity 55
13.14 Confidentiality 55

14. References 56

PROTOCOL SYNOPSIS

**Study Title** A Phase 3, Open-label, Multicenter Randomized Trial to Evaluate Humoral Immunogenicity of Various Schedules of Intramuscular Full Dose and Intradermal Fractional Doses of Inactivated Polio Vaccine in Latin American Infants			
**Product**	IPV and f-IPV	**Clinical Phase**	Phase 3
**Protocol Number**	IPV-004 ABMG	**Indication**	Injectable Polio Vaccine immunization
**ClinicalTrials.gov Number**	NCT03239496		

**Sponsor**	FIDEC
**Sponsor Representative**	Ricardo Rüttimann, MD
**Investigational Sites:** Hospital Maternidad Nuestra Señora de la Altagracia in Dominican Republic, and Cevaxin in Panamá City and Chiriquí	

Primary Objective:

1. To determine if the seroconversion rate of a 2-dose intradermally administered fractional-dose inactivated poliovirus vaccine (f-IPV) regimen administered at 14 and 36 weeks of age is non-inferior to that of a 2-dose intramuscularly administered inactivated poliovirus vaccine (IPV) regimen administered at 14 and 36 weeks of age 4 weeks after the last dose, for poliovirus serotypes 1 and 2.
2. To determine if the seroconversion rate of a 2-dose IPV regimen administered at 14 and 36 weeks of age is non-inferior to that of a 3-dose IPV regimen administered at 10, 14, and 36 weeks of age 4 weeks after the last dose, for poliovirus serotypes 1 and 2.
3. To determine if the seroconversion rate of a 2-dose f-IPV regimen administered at 14 and 36 weeks of age is non-inferior to that of a 3-dose f-IPV regimen administered at 10, 14, and 36 weeks of age 4 weeks after the last dose, for poliovirus serotypes 1 and 2.

Secondary Objectives:

1. To determine if the seroconversion rate of a 2-dose IPV regimen administered at 14 and 36 weeks of age is superior to that of a 2-dose IPV regimen administered at 10 and 14 weeks of age, 4 weeks after the second dose for poliovirus serotypes 1 and 2.
2. To determine if the seroconversion rate of a 2-dose f-IPV regimen administered at 14 and 36 weeks of age is superior to that of a 2-dose f-IPV regimen administered at 10 and 14 weeks of age 4 weeks after the second dose, for poliovirus serotypes 1 and 2.
3. To determine if the seroconversion rate of a 2-dose f-IPV regimen administered at 14 and 36 weeks of age is non-inferior to that of a 3-dose IPV regimen administered at 10, 14, and 36 weeks of age, 4 weeks after the last dose, for poliovirus serotypes 1 and 2.
4. To determine if the seroconversion rate of a 3-dose f-IPV regimen administered at 10, 14, and 36 weeks of age is non-inferior to that of a 3-dose IPV regimen also administered at 10, 14, and 36 weeks of age, 4 weeks after the last dose, for poliovirus serotypes 1 and 2.
5. To determine if the seroconversion rate to a 3-dose regimen of f-IPV administered at 10, 14, and 36 weeks of age is non-inferior to that of a 2-dose IPV regimen administered at 14 and 36 weeks of age, 4 weeks after the last dose for poliovirus serotypes 1 and 2.
6. To assess the safety of each vaccine (IPV and f-IPV) as measured by the incidence of serious adverse events (SAEs), important medical events (IMEs) and severe local reactions.
7. To assess all primary and secondary immunogenicity objectives through comparison of neutralizing antibody (NAb) titers.

Exploratory Objectives:

1. To assess immune responses to poliovirus type 3 analogous to the primary and secondary immunological objectives.
2. To determine if an additional dose of IPV at 36 weeks of age provides a superior humoral immune response when added to a 2-dose IPV regimen at 10 and 14 weeks of age, as compared to the 2-

dose regimen alone, for all three poliovirus serotypes, as measured by seroconversion rate, geometric mean and median NAb titers, 4 weeks after the last dose.

3. To determine if an additional dose of f-IPV at 36 weeks of age provides a superior humoral immune response when added to a 2-dose f-IPV regimen at 10 and 14 weeks of age, as compared to the 2-dose regimen alone, for all three poliovirus serotypes, as measured by seroconversion rate, geometric mean and median NAb titers, 4 weeks after the last dose.
4. To determine if the humoral immune response to a single dose of f-IPV administered at 10 weeks of age is non-inferior to that of a single dose of IPV administered at 10 weeks of age, for all three poliovirus serotypes, 4 weeks following vaccination, as measured by seroconversion rate, geometric mean and median NAb titers.
5. To determine if the humoral immune response to a 2-dose regimen of f-IPV administered at 10 and 14 weeks of age is non-inferior to that of a 2-dose regimen of IPV administered at 10 and 14 weeks of age, for all three poliovirus serotypes, as measured by seroconversion rate, geometric mean and median NAb titers, 4 weeks after the second dose.
6. To determine if the humoral immune response to a single dose of f-IPV administered at 14 weeks of age is non-inferior to that of a single dose of IPV administered at 14 weeks of age, for all three poliovirus serotypes, 4 weeks following vaccination, as measured by seroconversion rate, geometric mean and median NAb titers.
7. To determine if the humoral immune response to a single dose of f-IPV administered at 14 weeks of age is non-inferior to that of a single dose of IPV administered at 14 weeks of age, for all three poliovirus serotypes, 22 weeks following vaccination, as measured by seroconversion rate, geometric mean and median NAb titers.
8. To assess the response to an additional f-IPV dose administered at 36 weeks of age to infants receiving a single dose of f-IPV at 14 weeks of age, for all three poliovirus serotypes, as measured by seroconversion rate, geometric mean and median NAb titers, 4 weeks after the final dose.
9. To assess the response to an additional IPV dose administered at 36 weeks of age to infants receiving a single dose of IPV at 14 weeks of age, for all three poliovirus serotypes, as measured by seroconversion rate, geometric mean and median NAb titers, 4 weeks after the final dose.
10. To describe the relationship between the humoral immune response to vaccination, as measured by seroconversion rate and median NAb titers at weeks 18, 36 and 40, and each post-vaccination fluid bleb diameter in the group receiving two doses of ID f-IPV at weeks 14 and 36, for serotypes 1 and 2.

Primary Endpoints:

1. Serotypes 1 and 2 seroconversion four weeks after the second vaccination for the groups receiving 2 doses of f-IPV or 2 doses of IPV
2. Serotypes 1 and 2 seroconversion four weeks after the last vaccination for the group receiving 2 doses of IPV and for the group receiving 3 doses of IPV
3. Serotypes 1 and 2 seroconversion four weeks after the last vaccination for the group receiving 2 doses of f-IPV and for the group receiving 3 doses of f-IPV

Seroconversion will be defined as a change from seronegative to seropositive (antibody titers of ≥1:8), and in infants seropositive at baseline (assumed to be from maternally-derived antibody titers), as a ≥4-fold rise in antibody titers post-vaccination, computed by assuming an exponential decay model with a half-life of 24 days.

Secondary Endpoints:

In addition to primary endpoints:

1. Serotypes 1 and 2 seroconversion at week 40 for the groups receiving 2 doses of IPV at weeks 14 and 36 and at week 18 for the groups receiving 2 doses of IPV at weeks 10 and 14.
2. Serotypes 1 and 2 seroconversion at week 40 for the groups receiving 2 doses of f-IPV at weeks 14 and 36 and at week 18 for the groups receiving 2 doses of f-IPV at weeks 10 and 14.
3. Serotypes 1 and 2 seroconversion at week 40 for the group receiving 2 doses of f-IPV and the group receiving 3 doses of IPV.
4. Serotypes 1 and 2 seroconversion at week 40 for the groups receiving 3 doses of f-IPV or 3 doses of IPV.

5. Serotype 1 and 2 seroconversion at week 40, for the group receiving 3 doses of f-IPV, compared to the group receiving 2 doses of IPV at weeks 14 and 36
6. Safety Endpoints: SAEs, IMEs and severe local reactions throughout the study period.
7. Serotype 1 and 2 geometric mean and median NAb titers through all immunogenicity endpoints.

Exploratory Endpoints:

Humoral immune response defined as seroconversion to serotypes 1, 2 and 3, geometric mean and median NAb titers for the following endpoints, in addition to primary and secondary endpoints.

1. Humoral immune response to serotype 3, four weeks after each vaccination for all groups receiving IPV and f-IPV, and 22 weeks after receiving a single dose of IPV or f-IPV for the group receiving doses at weeks 14 and 36.
2. Humoral immune response at weeks 18 and 40, for the group receiving 3 doses of IPV
3. Humoral immune response at weeks 18 and 40, for the group receiving 3 doses of f-IPV
4. Humoral immune response at week 14, for the groups receiving 1 dose of f-IPV or 1 dose of IPV at week 10
5. Humoral immune response at week 18, for the groups receiving 2 doses of f-IPV or 2 doses of IPV at weeks 10 and 14
6. Humoral immune response at week 18, after receiving 1 dose of f-IPV or 1 dose of IPV at week 14
7. Humoral immune response at week 36, for the groups receiving 1 dose of f-IPV or 1 dose of IPV at week 14
8. Humoral immune response at week 40 for infants receiving 2 doses of f-IPV
9. Humoral immune response at week 40 for infants receiving 2 doses of IPV
10. Humoral immune response to serotypes 1 and 2, measured at weeks 18, 36 and 40 according to the diameter of fluid blebs (measured in millimeters) resulting from ID f-IPV vaccination at weeks 14 and 36, in the group receiving two doses of f-IPV

Overview of Study Design:

This will be an open-label, multicenter, randomized trial to be conducted in various pediatric centers in Dominican Republic and Panama. The subjects will be allocated to 4 study groups, in which an IPV vaccine with two different indications will be used:

- IPV full dose via intramuscular injection, or
- f-IPV fractional dose via intradermal administration

The vaccines will be administered in different immunization schedules to 773 healthy infants at different weeks of age, as shown in Table 1.

Table 1. Summary of study vaccine group assignments, administration of study vaccine and blood samples

Vaccine	Group*	Vaccination weeks**	Blood samples***	Subjects
**IM IPV**	A	10, 14, 36	10, 14, 18, 40	200
	B	14, 36	14, 18, 36, 40	178
**ID f-IPV§**	C	10, 14, 36	10, 14, 18, 40	178
	D	14, 36	14, 18, 36, 40	217
**Total**				773

*Groups A and C (3 doses), will also serve as 2-dose arms for 10 and 14 weeks regimens.
** Allowed windows: First and second dose administered at weeks 10 and/or 14 : +7 days
Second and third dose administered at week 36: -7+7 days
*** Allowed window for the blood samples: +7 days
Allowed window for blood samples at week 36: -7+7 days
§ Fractional-dose IPV Intradermal

Study Population:

The study will be conducted in various pediatric centers in Dominican Republic and Panama. The sites belong to both public and private health care providers. Parents or legal representatives of the infants will be invited to participate in the study at the earliest possible age. Infants will be considered eligible according to the following criteria:

Eligibility Criteria:

Inclusion Criteria

1. Infants of 6 weeks of age (-7 to + 7 days) on date of enrollment.
2. Healthy, as assessed from medical history and physical examination by a study physician,
3. Written informed consent obtained from parents or legal representatives who have been properly informed about the study and are able to comply with planned study procedures.

Exclusion Criteria

1. Vaccinated with any poliovirus vaccine prior to inclusion,
2. A household contact with OPV vaccination history in the past 4 weeks,
3. HIV infection or pharmacologic or clinical immunosuppression,
4. Known allergy to any component of the study vaccines (phenoxyethanol, formaldehyde),
5. Uncontrolled coagulopathy or blood disorder contraindicating intramuscular and intradermal injections,
6. Acute severe febrile illness on day of vaccination deemed by the Investigator(s) to be a contraindication for vaccination,
7. Not suitable for inclusion or is unlikely to comply with the protocol in the opinion of the investigator(s).

Study Procedures

An overview of the timing of vaccine administration and assessments is given in Table 2. Time and Events Schedule. SAEs, IMEs and severe local reactions will be assessed throughout the study participation.

Table 2. Time and Events Schedule

**Age of infants (weeks)**	**6 W**	**10 W**	**14 W**	**18 W**	**36 W**	**40 W**
**Visit ***	**Visit 1**	**Visit 2**	**Visit 3**	**Visit 4**	**Visit 5**	**Visit 6**
**Allowed visit window for vaccination and blood samples**	-7+7 days	+7 days	+7 days	+ 7 days	-7+7 days	+ 7 days
Informed Consent	X					
Inclusion/Exclusion criteria	X	X	X			
Medical history	X	X	X			
Physical examination	X	X	X		X	
Randomization and allocation	X					
Concomitant vaccines administration	X^{+}	X^{+}	X^{+}			
Vaccination Groups A/C		IPV/f-IPV	IPV/f-IPV		IPV/f-IPV	
Blood sample Groups A/C		X	X$^{\beta}$	X		X$^{\beta}$
Vaccination Groups B/D			IPV/f-IPV		IPV/f-IPV	
Blood sample Groups B/D			X	X$^{\beta}$	X	X$^{\beta}$
Check contraindications		X$^{@}$	X$^{@}$		X$^{@}$	
Immediate surveillance (30 mins) after vaccination		X$^{@}$	X$^{@}$		X$^{@}$	
Measurement of fluid bleb after ID vaccination for Group D $^{\mu}$		X	X			

Serious Adverse (SAEs), Important Medical Events (IMEs) and severe local reactions	*To be reported at any moment throughout the study*

* Not all groups will attend the same visits. Infants' attendance depends on their vaccination regimen
+ Concomitant vaccines at weeks 6 and 14 are Pneumococcal, Rotavirus and DTPw-HB-Hib and at week 10 only DTPw-HB-Hib
β Determination of hematocrit, hemoglobin and RBC morphology in the second and in the last blood sample
@ For study groups receiving vaccination
µ Measurement will be performed with a standard millimeter ruler

Infants from study groups B, C and D will receive 1 or 2 additional doses of full IPV intramuscularly after the end of the study. Thus, by 12 months of age, all children should be considered protected against all polioviruses.

Statistical Methods:

Sample size

It is assumed that almost all participants enrolled will provide data for safety analysis and at least 90 percent of enrolled subjects will be evaluable for immunogenicity assessments.

This study utilizes superiority tests (Fisher's exact test) and non-inferiority tests (score-based confidence intervals, non-inferiority margin = 10%) of seroconversion rates. Sample size calculations are based on assumed seroconversion rates that apply to all three serotypes. The seroconversion rates for all types after two doses of f-IPV are assumed to be 64% (10/14 regimen) or 94% (14/36 regimen) and after two doses of IPV are assumed to be 80% (10/14 regimen) or 96% (14/36 regimen).

There are two different power computations: the power of the comparison (either superiority or non-inferiority) to be successful for any individual serotype and the power to be successful for both serotypes 1 and 2 simultaneously, assuming independence of responses for each serotype.

The group sizes are chosen to provide ≥80% power for each of the primary objectives for the individual statistical tests of each serotype, as well for the test of both serotypes simultaneously. Power of approximately ≥80% is also available for all secondary objectives, except for the comparison of the f-IPV regimen administered at weeks 14 and 36 to the 3-dose IPV regimen, which has power for 62% for individual serotypes, and 39% for the combined serotypes. Given the much larger sample size necessary to boost the sample size to meet 80% power for this objective, this was considered an acceptable compromise for a feasible study designed mainly to assess primary objectives.

Based on the above, 773 infants will be included in the trial in order to account for an estimated ~10% drop-outs/withdrawals, for a total of 695 evaluable subjects.

Comparisons of geometric mean and median NAb titers between regimens will be conducted using geometric mean titer (GMT) ratios, facilitated by an analysis of covariance (ANCOVA) model of the $\log_2$ titer, which adjusts for the baseline level and study site as fixed effects. Non-inferiority of regimen 1 to regimen 2 will be declared if the lower 95% bound of the two-sided confidence interval for the GMT ratio (regimen 1 relative to regimen 2) is greater than 0.67. If an excessive amount of censoring at LLOQ or ULOQ occurs in either group, a one-sided randomization test for the median titer ratio, with resampling conducted within study site and using alpha level 0.025, will be applied instead. Details will be described in a statistical analysis plan, prepared prior to database lock and unblinding.

LIST OF ABBREVIATIONS AND DEFINITIONS OF TERMS

List of Abbreviations

AE	Adverse event
bOPV	Bivalent oral polio vaccine
CDC	Centers for Disease Control and Prevention
CI	Confidence interval
CRF	Case Report Form
EPI	WHO Expanded Programme on Immunization
f-IPV	Fractional-dose Inactivated polio vaccine
GCP	Good Clinical Practice
GMT	Geometric Mean Titers
GPEI	Global Polio Eradication Initiative
ICF	Informed Consent Form
ICH	International Council for Harmonization of Technical Requirements for Pharmaceuticals for Human Use
ID	Intradermal administration
IEC	Independent Ethics Committee
IM	Intramuscular administration
IME	Important medical event
IPV	Inactivated polio vaccine
IRB	Independent Review Board
ITT	Intention to treat
MedDRA	Medical Dictionary for Regulatory Activities
NIP	National Immunization Program
OPV	Oral polio vaccine
PAHO	Pan American Health Organization
PI	Principal Investigator
PP	Per protocol
SAE	Serious adverse event

SAGE	Strategic Advisory Group of Experts on Immunization
SAP	Statistical Analysis Plan
SC	Seroconversion
SII	*Serum Institute of India*
TAG	Technical Advisory Group on Vaccine-preventable Diseases
tOPV	Trivalent oral polio vaccine
TVP	Total vaccinated population
WHO	World Health Organization

STUDY ADMINISTRATIVE STRUCTURE

**SPONSOR**	FIDEC – Fighting Infectious Diseases in Emerging Countries (with grant support from the Bill and Melinda Gates Foundation)<br><br>2050 Coral Way, Suite 407<br>Miami, FL 33145 USA
Medical Expert:	Ricardo Rüttimann, MD
Biostatistician:	Christopher Gast, PhD
**MONITORING**	VAXTRIALS<br>Calle 50, FyF Tower, 12B, Panama City, Panama
Project Manager:	Jose Jimeno, MD

1. INTRODUCTION

1.1 BACKGROUND INFORMATION

In 2013, the Global Polio Eradication Initiative (GPEI) launched the Polio Eradication and Endgame Strategic Plan with the aim to end all polio disease globally. The four main objectives of the Polio Eradication and Endgame Strategic Plan are to detect and interrupt all poliovirus transmission, to strengthen immunization systems and withdraw the oral polio vaccine (OPV), to contain poliovirus and certify interruption of transmission, and transition planning.[1]

The global effort to eradicate polio has made significant progress so that only 3 countries remain where wild-type poliovirus transmission has never been interrupted —Afghanistan, Pakistan and Nigeria. [2]

For decades, trivalent oral polio vaccine (tOPV, with poliovirus types 1, 2, and 3) was the preferred vaccine for polio control and eradication. Global use of this vaccine has enabled the elimination of wild poliovirus type 2 and also contributed to the progress made in reducing the incidence of type 1 and 3 disease in most geographies.[3] However, in many developing countries, a reduced per dose immune response to poliovirus types 1 and 3 has been observed with tOPV[4]. The bivalent oral polio vaccine (bOPV), which does not contain type 2, is more effective against the types 1 and 3 wild poliovirus [5].

With the successful, globally synchronized switch from tOPV to bOPV in April 2016, IPV is currently the only source of type-2 immunity for polio.[6] Several clinical trials in recent years in the Latin America region, and elsewhere, have contributed to the clinical evidence base around such new b OPV – IPV mixed and sequential schedules that have been adopted globally.[7, 8, 9] Except for approximately 40 countries where IPV introduction had to be delayed due to supply constraints, most of the developing countries have included one dose of IPV in their bOPV primary series. The WHO, through the Strategic Advisory Group of Experts on Immunization (SAGE), and the PAHO, through the Technical Advisory Group (TAG), has stressed on the need to use alternative IPV vaccination schemes to cope with the constraints in the supply and has recommended intradermal application of fractional doses of IPV, instead of a single full dose of IPV, if needed[10,11, 12]. Also, it has become urgent to generate scientific evidence that allows to establish which reduced-dose or fractional dose IPV schemes would work optimally.

IPV is made from Salk (wild) strains or, less frequently, from Sabin (attenuated) strains and grown in Vero cell culture, or in human diploid cells (MRC-5). Manufacture of all current vaccines relies on inactivation of cell culture-derived polioviruses with formaldehyde in a final formulation containing standardized antigen units for each serotype. According to manufacturer specifications, IPV can be administered by subcutaneous or intramuscular injection. A fractional dose of stand-alone IPV can be administered intradermally.[13]

IPV is considered very safe, whether administered alone or in combination with other vaccines. There is no proven causal relationship with any adverse events other than temporary minor local symptoms such as erythema (<1%), induration (3%–11%) and tenderness (14%–29%).[14,15]

Studies have generally demonstrated that a single fractional dose of IPV (f-IPV 1/5 of the full dose) gives lower seroconversion rates than a full dose, but after 2 fractional doses, the

rates are similar to those achieved after 2 full doses. Studies in Cuba and in Bangladesh have shown 2 doses of f-IPV inducing seroconversion rates of 98% to poliovirus type 2 in Cuba at 4 and 8 months of age, and 81% to poliovirus type 2 in Bangladesh at 6 and 14 weeks of age. The results indicate that 2 fractional doses of IPV provide higher seroconversion rates than the achieved with a single full dose, being 63% at 4 months of age in Cuba[16] and 39% at 6 weeks of age in Bangladesh[17]. However, seroconversion is still inferior to that of 2 full doses of IPV[18]. This approach (2 fractional doses instead of 1 full dose), might increase the immune response as compared to a single dose and could extend coverage if supplies are limited. Moreover, intradermal administration of fractional doses of IPV (0.1 mL or 1/5 of a full dose) offers potential moderate cost reduction[18,19]; yet the cost and logistics of using this method still need to be considered[20].

Study Rationale

The rationale for this study (IPV-004 ABMG) is to assess and compare the immune response to full-dose inactivated polio vaccines (IPV) via intramuscular (IM) administration and of the fractional dose of inactivated poliovirus vaccine (f-IPV) via intradermal (ID) administration, in different schedule combinations in the EPI primary series.

This study prioritizes comparisons involving two-dose regimens recently recommended by the WHO Strategic Advisory Group of Experts on immunization (SAGE) and PAHO[11] in response to global IPV supply shortages [21]. Furthermore, the study will provide data on the comparative humoral immunogenicity of various schedules to inform polio immunization policy for the post-eradication era.

The study population will include infants in Dominican Republic and in Panama. Absence of wild and circulating vaccine derived polioviruses along with the lack of regular Supplementary Immunization Activities (SIAs) in the Latin America region provide an ideal epidemiologic setting to study polio vaccine immunogenicity.

Infants will receive two or three doses of full-dose IPV IM or f-IPV ID, in two schedules (10, 14 and 36 weeks and 14 and 36 weeks). Immunological and safety assessments will be made after one dose, two doses and three doses.

A total of 773 infants will be enrolled and distributed into 4 groups, according to a randomization scheme. During the study period, infants will be administered other concomitant vaccines according to the national schedules of the participating countries, but the effect, if any, of the concomitant administration on IPV immunogenicity will not be assessed.

Optimum immunogenicity expected from the dose(s) of IPV in the post-eradication era will have to be balanced with the cost and supply constraints of IPV. This study will be critical to determine how many doses of IPV and which schedule are optimal for the post-eradication era after the global cessation of OPV use.

1.2 RISK BENEFIT ANALYSIS

1.2.1 *Potential Risks*

The anticipated risks associated with the participation in the study are expected to be very low. The IPV vaccines to be studied are safe, effective, are licensed in Latin America, and have been used since the 1950s. All children would receive IPV as part of the NIP schedule.

As with all vaccines, allergic reactions of various severity may occur within a few minutes to a few hours after vaccination if the recipient is sensitive to components or excipients present in the vaccine product. As with all injectable vaccines, local injection site reactions of various severities may occur. Potential risks of venipuncture such as mild pain or hematoma are also expected to be low.

ID administration is being considered by the WHO and the PAHO as a recommendation to be evaluated in countries in situations of outbreaks or scarce vaccine supply. With intradermal vaccination, possible local reactions as redness, induration and infiltration may occur [22.] Depending on group assignment, some infants participating in the study will receive IPV or f-IPV vaccine just a few weeks later than they would otherwise receive them in the national immunization schedule of each country. Children which have received less than the recommended 3 full doses of IPV in the primary schedule will receive additional full IPV doses after the end of the study Thus, by 12 months of age, all children should be considered protected against all polioviruses.

Infants will be monitored at the study centers after vaccinations, and SAEs, IMEs and severe local reactions observed will be recorded in the infant's CRF. Furthermore, SAEs, IMEs and severe local reactions will be followed during the whole study period. Also, for the group receiving intradermal vaccination at weeks 14 and 36, the diameter of the bleb (if it occurs) will be measured in millimeters.

1.2.2 *Potential Benefits*

The outcome of this trial will provide the participating countries and the Latin American Region, as well as WHO SAGE, with information on vaccination policy development for the current setting of scarce IPV supply and for the OPV cessation era. It will also provide information on optimal number of doses and route of administration of IPV in the context of the post-eradication era, balancing immune response, risk of infection, supply constraints and cost. It will also help middle-and low-income countries to determine the best way to sustain polio eradication with the use of IPV. By strengthening the post-eradication immunization policy formulation, the study is expected to enhance the prospect of sustaining a polio-free world for long-term, and thereby protect each and every children globally from the paralytic disease.

2. STUDY OBJECTIVES

2.1 PRIMARY OBJECTIVES

1. To determine if the seroconversion rate of a 2-dose intradermally administered fractional-dose inactivated poliovirus vaccine (f-IPV) regimen administered at 14 and 36 weeks of age is non-inferior to that of a 2-dose intramuscularly administered inactivated poliovirus vaccine (IPV) regimen administered at 14 and 36 weeks of age 4 weeks after the last dose, for poliovirus serotypes 1 and 2.
2. To determine if the seroconversion rate of a 2-dose IPV regimen administered at 14 and 36 weeks of age is non-inferior to that of a 3-dose IPV regimen administered at 10, 14, and 36 weeks of age 4 weeks after the last dose, for poliovirus serotypes 1 and 2.
3. To determine if the seroconversion rate of a 2-dose f-IPV regimen administered at 14 and 36 weeks of age is non-inferior to that of a 3-dose f-IPV regimen administered at 10, 14, and 36 weeks of age 4 weeks after the last dose, for poliovirus serotypes 1 and 2.

2.2 SECONDARY OBJECTIVES

1. To determine if the seroconversion rate of a 2-dose IPV regimen administered at 14 and 36 weeks of age is superior to that of a 2-dose IPV regimen administered at 10 and 14 weeks of age, 4 weeks after the second dose for poliovirus serotypes 1 and 2.
2. To determine if the seroconversion rate of a 2-dose f-IPV regimen administered at 14 and 36 weeks of age is superior to that of a 2-dose f-IPV regimen administered at 10 and 14 weeks of age 4 weeks after the second dose, for poliovirus serotypes 1 and 2.
3. To determine if the seroconversion rate of a 2-dose f-IPV regimen administered at 14 and 36 weeks of age is non-inferior to that of a 3-dose IPV regimen administered at 10, 14, and 36 weeks of age, 4 weeks after the last dose, for poliovirus serotypes 1 and 2.
4. To determine if the seroconversion rate of a 3-dose f-IPV regimen administered at 10, 14, and 36 weeks of age is non-inferior to that of a 3-dose IPV regimen also administered at 10, 14, and 36 weeks of age, 4 weeks after the last dose, for poliovirus serotypes 1 and 2.
5. To determine if the seroconversion rate of a 3-dose regimen of f-IPV administered at 10, 14, and 36 weeks of age is non-inferior to that of a 2-dose IPV regimen administered at 14 and 36 weeks of age, 4 weeks after the last dose for poliovirus serotypes 1 and 2.
6. To assess the safety of each vaccine (IPV and f-IPV) as measured by the incidence of serious adverse events (SAEs), important medical events (IMEs) and severe local reactions.
7. To assess all primary and secondary immunogenicity objectives through comparison of neutralizing antibody (NAb) titers.

2.3 EXPLORATORY OBJECTIVES

1. To assess immune responses to poliovirus type 3 analogous to the primary and secondary immunological objectives.
2. To determine if an additional dose of IPV at 36 weeks of age provides a superior humoral immune response when added to a 2-dose IPV regimen at 10 and 14 weeks of age, as compared to the 2-dose regimen alone, for all three poliovirus serotypes, as measured by seroconversion rate, geometric mean and median NAb titers, 4 weeks after the last dose.
3. To determine if an additional dose of f-IPV at 36 weeks of age provides a superior humoral immune response when added to a 2-dose f-IPV regimen at 10 and 14 weeks of age, as compared to the 2-dose regimen alone, for all three poliovirus serotypes, as measured by seroconversion rate, geometric mean and median NAb titers, 4 weeks after the last dose.
4. To determine if the humoral immune response to a single dose of f-IPV administered at 10 weeks of age is non-inferior to that of a single dose of IPV administered at 10 weeks of age, for all three poliovirus serotypes, 4 weeks following vaccination, as measured by seroconversion rate, geometric mean and median NAb titers.
5. To determine if the humoral immune response to a 2-dose regimen of f-IPV administered at 10 and 14 weeks of age is non-inferior to that of a 2-dose regimen of IPV administered at 10 and 14 weeks of age, for all three poliovirus serotypes, as measured by seroconversion rate, geometric mean and median NAb titers, 4 weeks after the second dose.
6. To determine if the humoral immune response to a single dose of f-IPV administered at 14 weeks of age is non-inferior to that of a single dose of IPV administered at 14 weeks of age, for all three poliovirus serotypes, 4 weeks following vaccination, as measured by seroconversion rate, geometric mean and median NAb titers.
7. To determine if the humoral immune response to a single dose of f-IPV administered at 14 weeks of age is non-inferior to that of a single dose of IPV administered at 14 weeks of age, for all three poliovirus serotypes, 22 weeks following vaccination, as measured by seroconversion rate, geometric mean and median NAb titers.
8. To assess the response to an additional f-IPV dose administered at 36 weeks of age to infants receiving a single dose of f-IPV at 14 weeks of age, for all three poliovirus serotypes, as measured by seroconversion rate, geometric mean and median NAb titers, 4 weeks after the final dose.
9. To assess the response to an additional IPV dose administered at 36 weeks of age to infants receiving a single dose of IPV at 14 weeks of age, for all three poliovirus serotypes, as measured by seroconversion rate, geometric mean and median NAb titers, 4 weeks after the final dose.
10. To describe the relationship between the humoral immune response to vaccination, as measured by seroconversion rate and median NAb titers at weeks 18, 36 and 40, and each post-vaccination fluid bleb diameter in the group receiving two doses of ID f-IPV at weeks 14 and 36, for serotypes 1 and 2.

3. STUDY ENDPOINTS

3.1 PRIMARY ENDPOINTS

1. Serotypes 1 and 2 seroconversion four weeks after the second vaccination for the groups receiving 2 doses of f-IPV or 2 doses of IPV
2. Serotypes 1 and 2 seroconversion four weeks after the last vaccination for the group receiving 2 doses of IPV and for the group receiving 3 doses of IPV
3. Serotypes 1 and 2 seroconversion four weeks after the last vaccination for the group receiving 2 doses of f-IPV and for the group receiving 3 doses of f-IPV

Seroconversion will be defined as a change from seronegative to seropositive (antibody titers of ≥1:8), and, in infants seropositive at baseline (assumed to be from maternally-derived antibody titers), as a ≥4-fold rise in antibody titers post-vaccination, computed by assuming an exponential decay model with a half-life of 24 days.

3.2 SECONDARY ENDPOINTS

In addition to primary endpoints:

1. Serotypes 1 and 2 seroconversion at week 40 for the groups receiving 2 doses of IPV at weeks 14 and 36 and at week 18 for the groups receiving 2 doses of IPV at weeks 10 and 14.
2. Serotypes 1 and 2 seroconversion at week 40 for the groups receiving 2 doses of f-IPV at weeks 14 and 36 and at week 18 for the groups receiving 2 doses of f-IPV at weeks 10 and 14.
3. Serotypes 1 and 2 seroconversion at week 40 for the group receiving 2 doses of f-IPV and the group receiving 3 doses of IPV.
4. Serotypes 1 and 2 seroconversion at week 40 for the groups receiving 3 doses of f-IPV or 3 doses of IPV.
5. Serotype 1 and 2 seroconversion at week 40, for the group receiving 3 doses of f-IPV, compared to the group receiving 2 doses of IPV at weeks 14 and 36
6. Safety Endpoints: SAEs, IMEs and severe local reactions throughout the study period.
7. Serotype 1 and 2 geometric mean and median NAb titers through all immunogenicity endpoint

3.3 EXPLORATORY ENDPOINTS

Humoral immune response defined as seroconversion to serotypes 1, 2 and 3, geometric mean and median Nab titers for the following endpoints, in addition to primary and secondary endpoints.

1. Humoral immune response to serotype 3, four weeks after each vaccination for all groups receiving IPV and f-IPV, and 22 weeks after receiving a single dose of IPV or f-IPV for the group receiving doses at weeks 14 and 36.
2. Humoral immune response at weeks 18 and 40, for the group receiving 3 doses of IPV
3. Humoral immune response at weeks 18 and 40, for the group receiving 3 doses of f-IPV
4. Humoral immune response at week 14, for the groups receiving 1 dose of f-IPV or 1 dose of IPV at week 10

5. Humoral immune response at week 18, for the groups receiving 2 doses of f-IPV or 2 doses of IPV at weeks 10 and 14
6. Humoral immune response at week 18, after receiving 1 dose of f-IPV or 1 dose of IPV at week 14
7. Humoral immune response at week 36, for the groups receiving 1 dose of f-IPV or 1 dose of IPV at week 14
8. Humoral immune response at week 40 for infants receiving 2 doses of f-IPV
9. Humoral immune response at week 40 for infants receiving 2 doses of IPV
10. Humoral immune response to serotypes 1 and 2, measured at weeks 18, 36 and 40 according to the diameter of fluid blebs (measured in millimeters) resulting from ID f-IPV vaccination at weeks 14 and 36, in the group receiving two doses of f-IPV

4. STUDY DESIGN

4.1 OVERVIEW OF STUDY DESIGN

Overview of Study Design:

This will be an open-label, multicenter, randomized trial to be conducted in various pediatric centers in Dominican Republic and Panama. The subjects will be allocated to 4 study groups, in which an IPV vaccine with two different indications will be used:

- IPV using full dose via intramuscular injection
- f-IPV using fractional dose via intradermal administration

The vaccines will be administered in different immunization schedules to 773 healthy infants at different weeks of age, as shown in Table 1.

Table 1 Summary of study vaccine group assignments, administration of study vaccine and blood samples

Vaccine	Group	Vaccination weeks**	Blood samples***	Subjects
**IM IPV**	A	10, 14, 36	10, 14, 18, 40	200
	B	14, 36	14, 18, 36, 40	178
**ID f-IPV§**	C	10, 14, 36	10, 14, 18, 40	178
	D	14, 36	14, 18, 36, 40	217
**Total**				773

*Groups A and C (3 doses), will also serve as 2-dose arms for 10 and 14 weeks.
** Allowed windows: First and second dose administered at weeks 10 and/or 14: +7 days
Second and third dose administered at week 36: -7+7 days
*** Allowed window for the blood samples: +7 days
Allowed window for blood samples at week 36: -7+7 days
§ Fractional-dose IPV Intradermal

4.2 DISCUSSION OF STUDY DESIGN

This will be an open-label study. The study staff, including the one performing vaccination at the investigational site and the participants will know which vaccine and indication will be administered. However, the identity of the vaccine and indication administered will remain unknown to the staff performing the polio antibody analysis. Participants will be assigned to 4 different groups which will receive an IPV vaccine in a two- or three-dose regimen and two different indications: via intramuscular injection or via intradermal administration.

This design will allow use of data collected after the first, second and third doses from infants allocated to the two or three-dose regimens of the vaccines evaluated, to perform comparisons among the different study schedules.

5. SELECTION OF STUDY POPULATION

5.1 INCLUSION CRITERIA

The study will be conducted in outpatient pediatric centers and at private and public vaccination centers, depending on each country. Parents or legal representatives of the infants will be invited to participate in the study at the earliest possible age. Infants will be considered eligible according to the following criteria:

1. Infants of 6 weeks of age (-7 to + 7 days) on date of enrollment.
2. Healthy, as assessed from medical history and physical examination by a study physician.
3. Written informed consent obtained from parents or legal representatives that they have been properly informed about the study and are able to comply with planned study procedures.

5.2 EXCLUSION CRITERIA

1. Vaccinated with any poliovirus vaccine prior to inclusion.
2. A household contact with OPV vaccination history in the past 4 weeks.
3. HIV infection or pharmacologic immunosuppression.
4. Known allergy to any component of the study vaccines (phenoxyethanol, formaldehyde).
5. Uncontrolled coagulopathy or blood disorder contraindicating intramuscular and intradermal injections.
6. Acute severe febrile illness on day of vaccination deemed by the Investigator(s) to be a contraindication for vaccination.
7. Not suitable for inclusion or is unlikely to comply with the protocol in the opinion of the investigator(s).

5.3 CRITERIA FOR ELIMINATION FROM THE PER-PROTOCOL POPULATION

- Infant did not receive all vaccinations per protocol schedule,
- Violation of any inclusion /exclusion criteria
- A household contact with OPV vaccination history

5.4 CONTRAINDICATIONS TO FURTHER VACCINATION

Before administration of each dose of the study vaccine, permanent contraindications must be checked. The following adverse events (AEs) constitute absolute contraindications to further administration of the study vaccines:

- SAE, IME and severe local reactions, considered to be consistent with a causal association to the study vaccine (after first or second dose)
- Known hypersensitivity to any component of the vaccine or severe reaction following administration of the vaccine (after first or second dose)
- Acute severe febrile illness on day of vaccination deemed by the Investigator(s) to be a temporal contraindication for vaccination.
- Any intercurrent medical condition that in the judgment of the study physician will interfere with scheduled vaccinations and/or possibly impair the immune response to polio vaccination including those listed in Section 5.2

If any of these AEs occur during the study, the infant should not receive additional doses of the vaccine but may continue other study procedures at the discretion of the Investigator(s), including blood sampling. The infant will be followed until resolution of the event and to determine the immune response. Since vaccines to prevent these diseases are recommended for all infants, subsequent vaccination will be conducted after consultation with an appropriate sub-specialist to determine the most appropriate vaccines to administer.

6. VACCINES

6.1 PHYSICAL DESCRIPTION OF THE STUDY VACCINE

Inactivated Polio Vaccine

Poliomyelitis Vaccine (Inactivated), produced by Serum Institute of India (SII) Pvt. Ltd., and prequalified by WHO, is a sterile suspension of three types of inactivated poliovirus: Type 1 (Mahoney), Type 2 (MEF-1), and Type 3 (Saukett). Each of the three strains of poliovirus is individually grown in Vero cells, a continuous line of monkey kidney cells cultivated on microcarriers. Polio vaccine is manufactured from the bulk imported from Bilthoven Biologicals B.V., Bilthoven, The Netherlands. The vaccine meets the requirements of WHO when tested by the methods outlined in the current WHO requirements.

Each dose of vaccine (0.5 mL) contains 40 D antigen units of Mahoney strain (Type 1); 8 D antigen units of MEF-1 strain (Type 2); and 32 D antigen units of Saukett strain (Type 3). It also contains 2.5 mg of 2-phenoxyethanol and a maximum of 12.5 mcg of formaldehyde as preservatives

6.2 DOSE AND ADMINISTRATION

The dose of IPV vaccine should be 0.5 mL administered strictly intramuscularly in the anterolateral aspect of the thigh, using a standard needle and syringe.

The fractional IPV (f-IPV) dose should be 0.1 mL or 1/5 of the full IPV dose, and the administration site is strictly the upper arm, using a 0.1 mL AD (auto disable) syringe [23]. Care should be taken to avoid administering the injection into or near blood vessels and nerves.

IPV and f-IPV vaccines will be administered according to the study groups described in Table 1.

After administration of the study vaccines, the infant will be observed for at least 30 minutes for signs of any reaction, including measurement of the fluid bleb, if any, in infants receiving the intradermal vaccination at weeks 14 and 36.

6.3 OTHER MEDICATION ADMINISTERED IN THE STUDY

The following concomitant vaccines DTPw-HB-Hib, Pneumococcal and Rotavirus will be administered in the opposite thigh to which IPV will be applied, according to the schedule presented in Table 2.. All details from concomitants vaccines administered will be recorded in the CRF.

There will be no restrictions in using medications/treatments. Only medications to treat SAEs, IMEs and severe local reactions will be documented in the CRF. All other medications will only be captured in the source documentation at the investigational site.

6.4 PACKAGING AND LABELING

The Poliomyelitis Vaccine (Inactivated) is manufactured by Serum Institute of India and filled into multi-dose vials of up to 1mL (2-dose vial) or 2.5 mL (5-dose vial). The label and packaging will be according to WHO standards. The expiry date of the vaccine is indicated on the label and packaging.

Vaccine Vial Monitor (VVMs) are part of the label on Poliomyelitis Vaccine (Inactivated) supplied through Serum Institute of India Pvt. Ltd. The color dot which appears on the label of the vial is a VVM. This is a time-temperature sensitive dot that provides an indication of the cumulative heat to which the vial has been exposed. It warns the end user when exposure to heat is likely to have degraded the vaccine beyond an acceptable level

6.5 STORAGE AND VACCINE ACCOUNTABILITY

The Investigator(s) (or their designee) is responsible for the safe storage of all study vaccine assigned to the clinical site, in a locked, secure storage facility with access limited to those individuals authorized to dispense the study vaccine, and maintained within the appropriate ranges of temperature. In case the country does not have an IPV stand alone vaccine available, the Sponsor will supply it. All study vaccines must be stored as specified at delivery and in the original packaging. The IPV vaccine is stable if stored in the refrigerator at 2°C to 8°C. The vaccine must not be frozen.

Regular temperature logging of the refrigerator where the study vaccine will be stored at the clinical site should be performed. In case a deviation in storage conditions should occur, the clinical site must not further dispense the affected study vaccine and should notify the Sponsor.

The Investigator(s) will be responsible for ensuring that all study vaccines received at the clinical site are inventoried and accounted for throughout the study.

Study vaccine should be dispensed under the supervision of the Investigator(s), a qualified member of the clinical staff, or by a hospital/clinic pharmacist. The Investigator(s) must maintain accurate records demonstrating date and amount of vaccine administered to whom and by whom. Study vaccine will be supplied only to subjects participating in the study.

The Sponsor’s designated site monitor will periodically check the supplies of study vaccine held by the Investigator(s) or pharmacist to ensure accountability and appropriate storage conditions of all study vaccine used.

Unused study vaccine must be available for verification by the site monitor during on-site monitoring visits.

After the last visit of the last subject in the study (LSLV), any remaining study vaccine will be returned to the Sponsor, or destroyed at the clinical site with the Sponsor’s written permission (in this case a certificate of destruction will be provided and filed in the Trial Master File [TMF]).

6.6 RANDOMIZATION

Eligible infants will be randomized into one of the 4 study groups using computer-generated block randomization to balance allocation across sites. The allocations will be provided to the study Investigator(s) by a central location after informed consent has been obtained.

6.7 COMPLIANCE

Adequate and sufficient procedures will be established to ensure that the development of the clinical study is carried out in compliance with the global recommendations on Good

Clinical Practices and local regulations for clinical trials, ensuring adherence to the ethical principles of biomedical research and protection of the study population.

Subject compliance will be addressed by evaluating the vaccine administration data recorded by the staff in the study documents. During the clinical study, the monitor will check that vaccine administration data is recorded correctly.

7. PRIOR AND CONCOMITANT THERAPY

In addition to the study vaccines (IPV or f-IPV), participating infants will receive the DTPw-HB-Hib, Pneumococcal and Rotavirus concomitant vaccines according to the schedule presented in Table 2.

All concomitant intramuscular vaccines will be administered in the thigh or arm opposite to which IPV and f-IPV will be applied.

8. ASSESSMENTS

8.1 TIMING OF ASSESSMENTS

An overview of the timing of vaccine administration and assessments is given in Table 2.

Parent(s)/legal representatives will be given a full explanation of the nature of the study and written informed consent (approved by the local ethics committee) will be obtained from parent(s)/legal representatives according to local requirements before any study-related assessment will be carried out.

Serious Adverse Events (SAEs) and Important Medical Events (IMEs), severe local reactions and the diameter of the fluid bleb, if any, in infants who will receive intradermal administration at weeks 14 and 36, will be monitored after the first vaccination and until the last study-related activity.

A blood sample will be taken from infants according to their study group visit schedule. When it is a vaccination visit, blood sample will be taken before vaccination. Infants will receive the first dose of study vaccine according to the procedure described in Section 6.6, followed by further assessments as outlined in Table 2. A measurement of the fluid bleb after intradermal vaccination will be performed with a millimeter ruler. Infants will be kept under medical supervision for at least 30 minutes after each IPV and f-IPV vaccination.

Unscheduled visits can be planned; for instance:

- To obtain additional information to ensure safety to the infant. Additional blood and urine samples may be taken at the discretion of the Investigator(s).

Findings made during unscheduled visits should be reported in the source documents and in the designated sections of the eCRF.

Table 2 Time and Events Schedule

**Age of infants (weeks)**	**6 W**	**10 W**	**14 W**	**18 W**	**36 W**	**40 W**
**Visit ***	**Visit 1**	**Visit 2**	**Visit 3**	**Visit 4**	**Visit 5**	**Visit 6**
**Allowed visit window for vaccination and blood samples**	-7+7 days	+7 days	+7 days	+ 7 days	-7+7 days	+ 7 days
Informed Consent	X					
Inclusion/Exclusion criteria	X	X	X			
Medical history	X	X	X			
Physical examination	X	X	X		X	
Randomization and allocation	X					
Concomitant vaccines administration	X+	X+	X+			
Vaccination Groups A/C		IPV/f-IPV	IPV/f-IPV		IPV/f-IPV	
Blood sample Groups A/C		X	Xβ	X		Xβ
Vaccination Groups B/D			IPV/f-IPV		IPV/f-IPV	
Blood sample Groups B/D			X	Xβ	X	Xβ
Check contraindications		X@	X@		X@	

Immediate surveillance (30 mins) after vaccination		X@	X@		X@	
Measurement of fluid bleb after ID vaccination forGroup D µ			X		X	
Serious Adverse (SAEs), Important Medical Events (IMEs) and severe local reactions	*To be reported at any moment throughout the study*					

* Not all groups will attend the same visits. Infants' attendance depends on their vaccination regimen
+ Concomitant vaccines at weeks 6 and 14 are Pneumococcal Rotavirus and DTPw-HB-Hib and at week 10 only DTPw-HB-Hib
β Determination of hematocrit, hemoglobin and RBC morphology in the second and in the last blood sample
@ For study groups receiving vaccination
µ Measurement will be performed with a standard millimeter ruler

Infants from study groups B, C and D will receive 1 or 2 additional doses of full IPV intramuscularly after the end of the study. Thus, by 12 months of age, all children should be considered protected against all polioviruses.

8.1.1 *Collection and handling of blood samples*

A total of four blood samples will be collected from each infant, prior to vaccination and according to their scheduled visits. Blood will be collected using butterfly needle or hypodermic needles and syringe. A minimum of 3.5 mL and a maximum of 4 mL will be collected in each visit, for a total of approximately 16mL over the entire study period. Blood will be collected into blood tubes in the vaccination sites and then centrifuged to separate serum. Serum will be transferred to cryotubes suitable for serum collection and will be stored at -20°C until its transportation to the CDC designated laboratory. The shipping courier will be International Air Transport Association certified and arranged locally at each site. Procedures for processing blood samples will be conducted following internal standard operating procedures (SOPs) and laboratory manuals approved by the sponsor.

8.2 IMMUNOGENICITY

8.2.1 *Immunogenicity Variables*

Immunogenicity endpoints will be determined by assessment of neutralizing antibody titers against polioviruses 1, 2 and 3. The level of neutralizing antibody present on serum samples will be expressed as a titer. A serum -neutralization assay will be used for antibody determinations[24]

Type-specific neutralizing antibody titers will be summarized descriptively on the logarithmic (base 2) scale.

Seroprotection will be tabulated at each pre- and post-vaccination serology collection time point.

Seroconversion will be defined as a change from seronegative to seropositive (antibody titers of ≥1:8) and in infants seropositive at baseline (assumed to be from maternally-

derived antibody titers), as a ≥4-fold rise in antibody titers post-vaccination, computed by assuming an exponential decay model with a half-life of 24 days.

8.3 SAFETY EVALUATIONS

Safety will be evaluated using the following parameters:

- SAEs as defined in the protocol throughout the study period.
- IMEs and severe local reactions, as defined in the protocol throughout the study period.

8.3.1 *Serious Adverse Events*

The Investigator(s) will be responsible for recording and reporting to the pharmacovigilance reporting system and regulatory authorities in the participating countries, within 24 hours and according to regulatory timelines, all SAEs observed during the study (treatment and follow-up) period.

A SAE, experience or reaction, is any untoward medical occurrence (whether considered to be related to study vaccine or not) that at any dose:

- Results in death.
- Is life-threatening (the infant is at a risk of death at the time of the event; it does not refer to an event which hypothetically might have caused death if it were more severe).
- Requires inpatient hospitalization or prolongation of existing hospitalization: Hospital admissions and/or surgical operations planned before or during a study are not considered SAEs if the illness or disease existed before the infant was enrolled in the study, provided that it did not deteriorate in an unexpected way during the study.
- Results in persistent or significant disability/incapacity.
- Is a congenital abnormality/birth defect detected only after study inclusion.

8.3.2 *Important medical events (IMEs)*

IMEs are medically significant events that do not meet any of the SAE criteria above, but require medical or surgical consultation or intervention to prevent this event from becoming one of the serious outcomes listed in the SAE definition above.

Examples of important medical events include allergic bronchospasm requiring intensive treatment in an emergency room or at home, blood dyscrasias (e.g., neutropenia or anemia requiring blood transfusion, etc.).

To avoid judgement bias, IMEs will be reported separately and not as a subgroup of SAEs. They will be processed in the same way as SAEs. Every aspect described for SAEs (including trial objectives and endpoints) also applies to an IME.

The term "severe" is often used to describe the intensity (severity) of a specific event. The event itself, however, may be of relatively minor medical significance (such as severe headache). This is not the same as "serious," which is based on patient/event outcome or action criteria usually associated with events that pose a threat to a patient's life or functioning.

Severe local reactions can include severe pain, inflammation, induration and edema in the injection area[25]. Very rarely, abscess at injection site within 7 days of vaccine administration[26]. Nodules at the injection site with more than 2.5 centimeters in diameter and cellulitis[27].

9. STUDY TERMINATION/COMPLETION

9.1 STUDY COMPLETION

9.1.1 *Subject Completion*

Infants will complete the study four weeks after the last study vaccination, which is at week 40 for all study groups.

9.1.2 *Study Completion Date*

The study completion date is considered to be the date on which the final serologic analysis is available for the purpose of assessing the primary immunogenicity objective.

9.2 REMOVAL OF SUBJECTS FROM STUDY OR INVESTIGATIONAL PRODUCT

9.2.1 *Removal from Study*

Parents(s)/legal representatives have the right to withdraw their infants from the study at any time for any reason, including personal reasons. An infant can be withdrawn without giving a reason. The Investigator(s) should however try to find out why an infant is withdrawn from the study and document the reason for withdrawal in the source documents and on the eCRF.

Withdrawal will not affect in any way the treatment of the infant by the health care system. If the child is withdrawn, investigators will ensure that the child continues with their vaccination schedule according to the NIP in the participant countries.

Infants **may** be withdrawn from the study in the event of:

- A SAE, an IME or severe local reactions
- Difficulties in obtaining blood
- Failure of the subject and/or subject's parent(s)/legal representative to comply with the protocol requirements or to cooperate with the Investigator(s).

Infants **must** be withdrawn from the study in the event of:

- Administration of any poliovirus vaccines outside the study protocol
- Withdrawal of consent by parent(s)/legal representative;
- For safety reasons, if, in the Investigator's opinion, in the best interest of the infant.

In the event of an infant being withdrawn from the study, the monitor and Sponsor should be informed: in the event of withdrawal due to an SAE (for details on SAE reporting see Section 11), the Sponsor should be notified within 24 hours; in the event of withdrawal for other reasons, the Sponsor should be notified within 2 days from the event.

If there is a medical reason for withdrawal, the infant will remain under the supervision of the Investigator(s) until satisfactory health has returned.

Infants who are withdrawn from the study prior to completion of the scheduled study procedures for any reason (e.g. AE, withdrawal of consent) should be invited to complete the assessments as much as possible: as long as the infants' parent(s)/legal representative consents, all relevant assessments of the day on which the infant withdrew from the study

should be completed, at least those related to safety. In case of a SAE, the appropriate follow-up will be done.

Infants who are withdrawn from the study will not be replaced.

10. STATISTICAL METHODS

10.1 STATISTICAL ANALYSIS

All statistical methods shall be detailed in the Statistical Analysis Plan (SAP) that will be finalized before database lock.

Unless otherwise specified, descriptive statistics include mean, standard deviation (SD), median, maximum, minimum and range for continuous variables and the number and percentage in each group for categorical variables.

Unless specified otherwise in the SAP, statistical tests and confidence intervals (CIs) will be computed using a two-sided 5% significance level. P-values will be presented with 4 decimal places; p-values < 0.0001 will be presented as "< 0.0001".

Prior and concomitant medications will be coded using the WHO_DRUG Dictionary, and serious adverse events will be coded into system organ class and preferred term using MedDRA.

10.1.1 *Study Populations*

The following populations will be considered for analysis:

- Intention-to-treat (ITT) population, defined as all enrolled infants scheduled to receive study vaccine, regardless of whether a study vaccine was administered.
- Total Vaccinated population (TVP), defined as all infants who are in the ITT population who received at least one dose of study vaccine. Dropout from ITT to TVP will be described.
- Per-protocol (PP) population, consisting of all eligible study participants who are in the Total Vaccinated population who receive all of the immunizations scheduled for the group to which they are allocated and who do not meet any of the criteria in Section 5.3

Unless specified otherwise, the Total Vaccinated population will be used for safety analysis, and the PP population will be used for immunogenicity analysis. All immunogenicity analyses (primary and secondary) will be repeated in the TVP. Descriptive summaries of demographics will be computed in both the TV and PP populations.

10.1.2 *Initial Characteristics of the Subject Sample*

Unless otherwise specified, descriptive statistics will be provided per group for demographics (e.g., age, weight, race, gender), baseline seroprotection rates for each of the polioviruses, and other initial subject characteristics (e.g., medical and surgical history, concomitant diseases). Descriptive statistics include mean, standard deviation (SD), median, maximum, minimum and range for continuous variables, and the number and percentage in each group for categorical variables.

10.1.3 *Immunogenicity Data*

For an overview of primary, secondary and exploratory endpoints, see Section 3.

Poliovirus Antibody Titers

At each pre-and post-vaccination time point where neutralizing antibody titers are obtained:

- Seroprotection rates with 95% score-based confidence intervals (CIs) will be tabulated.
- Seroconversion rates with 95% score-based confidence intervals (CIs) will be computed at post-vaccination time points.
- Continuous summaries of $\log_2$ antibody titers will be computed along with 95% CIs for the median and geometric mean. CIs for the median will be obtained via the bootstrap method, and normal-based methods will be used for the geometric mean.
- Plots of the reverse cumulative distribution of antibody titers will be generated.

10.1.4 *Safety Data*

Safety will be assessed through collection and tabulation by study arm of Adverse Events.

Adverse events will be classified into standardized terminology from the verbatim description according to the most recent version of the MedDRA adverse event coding dictionary available. Adverse events will be presented by preferred term nested within system organ class. Verbatim description and all MedDRA-coded terms for all AEs will be contained in listings.

Adverse Events will be summarized by the incidence of AEs by body system and preferred term. The incidence of AEs will be based on the number and percent of subjects with AEs. Although a term may be reported more than once for a subject, that subject will be counted only once in the incidence count for that MedDRA term. The severity of the AEs and the causal association to study vaccine will be summarized for each body system and preferred term by group. Withdrawals due to AEs will be summarized for each body system and preferred term. Concomitant vaccines will be tabulated by study groups.

10.1.5 *Missing Data*

The reasons for any missing data will be ascertained and appropriate statistical methods will be used to accommodate these absences in the analyses of trial data, to minimize potential biases and maximize efficiency conditional on the causes for data being missing. Data values that are identified to be spurious by quality control procedures will not be used in final analyses of trial data. If an excessive amount of missing immunogenicity is present, additional sensitivity analyses may be computed to determine its influence on important analyses.

10.2 DETERMINATION OF SAMPLE SIZE

A total of 773 infants will be enrolled in the study. For sample size calculations, the assumed seroconversion rates applying to all three serotypes are presented in Table 3.

Table 3 Assumed SC rates at 4 weeks post last dose for poliovirus types 1, 2 and 3 after two doses of IPV, two doses of f-IPV, three doses of IPV and three doses of f-IPV

Group	Assumed SC rates at 4 weeks, post 2 doses	Assumed SC rates at 4 weeks, post 3 doses	Min N evaluable sample size	N for enrollment*
Group A	80%	99%	180	200
Group B	96%	NA	160	178
Group C	64%	96%	160	178
Group D	94%	NA	195	217
**Total**			**695**	**773**

* Assumes 10% dropout rate from enrolled population to PP population, for immunogenicity objectives

Power is computed for the primary and secondary seroconversion objectives. There are two different power estimates for the study: (Table 4)

1. The power of the comparison (either superiority or non-inferiority) to be successful for *any individual serotype.*
2. The power of the comparison (either superiority or non-inferiority) to be successful for *serotypes 1 and 2 simultaneously*

Table 4 Power estimation

Comparison*	Comparison Type Superiority vs. Non-Inferiority	Objective	Power for Any Individual Serotype	Power for both Serotypes Simultaneously
D vs B (2 f-IPV vs IPV at 14, 36)	NI	Primary	90%	80%
B vs A (IPV at 14/36 vs 10, 14, 36)	NI	Primary	90%	81%
D vs C (f-IPV at 14, 36 vs 10, 14, 36)	NI	Primary	90%	81%
Superiority of 14, 36 to 10, 14 (IPV)	S	Secondary	100%	99%
Superiority of 14, 36 to 10, 14 (f-IPV)	S	Secondary	100%	100%
D vs A (f-IPV at 14, 36 vs IPV at 10, 14, 36)	NI	Secondary	62%	39%
A vs C (3 f-IPV vs 3 IPV)	NI	Secondary	90%	80%
C vs B (3 f-IPV vs 2 IPV at 14, 36)	NI	Secondary	98%	96%

***** Comparisons to be made with study data. In general, groups A and C (3 doses) serve as 2 dose arms for 10 and 14 weeks represents

The group sizes, selected via simulation of the study using the assumed seroconversion rates and the efficacy analysis methods described below, are chosen to provide ≥ 80% power for each of the primary objectives, for the individual statistical tests of each serotype, as well as across both serotypes simultaneously. Power of ≥80% is also available for all secondary objectives, except for the comparison of the f-IPV regimen administered at weeks 14 and 36 to the 3-dose IPV regimen, which has power of 62% for individual serotypes, and 39% for the combined serotypes. Given the much larger sample size necessary to boost the sample size to meet 80% power for this objective, this was considered an acceptable compromise for a feasible study designed mainly to assess primary objectives. In order to conclude a vaccination schedule is completely

superior/non-inferior to another across both serotypes, the conclusion must be met for each serotype individually.

10.3 IMMUNOGENICITY ANALYSIS

A number of comparisons of seroconversion rates between vaccine regimens and vaccine types are intended to be made. All non-inferiority comparisons of seroconversion rates will be made utilizing the lower bound of two-sided score-based confidence intervals ($\alpha = 0.05$) with non-inferiority margin 10%, and superiority comparisons of seroconversion rates will be made using one-sided Fisher's exact test ($\alpha = 0.025$).

For all comparisons, type-specific superiority or non-inferiority of seroconversion rates will be presented; uniform superiority/non-inferiority will be declared if the superiority or non-inferiority conclusion can be made for both serotypes 1 and 2 simultaneously.

Comparisons of NAb titers between regimens will be conducted using geometric mean titer (GMT) ratios, facilitated by an analysis of covariance (ANCOVA) model of the $\log_2$ titer, which adjusts for the baseline level and study site as fixed effects. Non-inferiority of regimen 1 to regimen 2 will be declared if the lower 95% bound of the two-sided confidence interval for the GMT ratio (regimen 1 relative to regimen 2) is greater than 0.67. If an excessive amount of censoring at LLOQ or ULOQ occurs in either group, a one-sided randomization test for the median titer ratio, with resampling conducted within study site and using alpha level 0.025, will be applied instead.

Detailed specification of statistical methods and exploratory analyses will be fully-described in the Statistical Analysis Plan.

11. SERIOUS ADVERSE EVENT REPORTING

11.1 DEFINITIONS

11.1.1 *Serious Adverse Events*

The Investigator(s) will be responsible for recording and reporting within 24 hours and according to regulatory timelines all SAEs observed during the study (treatment and follow-up) period.

A SAE, experience or reaction, is any untoward medical occurrence (whether considered to be related to study drug or not) that at any dose:

- Results in death.
- Is life-threatening (the infant is at a risk of death at the time of the event; it does not refer to an event which hypothetically might have caused death if it were more severe).
- Requires inpatient hospitalization or prolongation of existing hospitalization: Hospital admissions and/or surgical operations planned before or during a study are not considered AEs if the illness or disease existed before the infant was enrolled in the study, provided that it did not deteriorate in an unexpected way during the study.
- Results in persistent or significant disability/incapacity.
- A congenital abnormality/birth defect will be considered only if it is detected after the inclusion of the infant in the study.

11.1.2 *Important Medical Event*

The Investigator(s) will be responsible for recording and reporting within 24 hours and according to regulatory timelines all IMEs observed during the study (treatment and follow-up) period.

Important medical events (IMEs) are medically significant events that do not meet any of the SAE criteria above, but require medical or surgical consultation or intervention to prevent this event to become one of the serious outcomes listed in the SAE definition above. Examples of important medical events include allergic bronchospasm requiring intensive treatment in an emergency room or at home, blood dyscrasias (e.g., neutropenia or anemia requiring blood transfusion, etc.) or convulsions that do not result in hospitalization. To avoid judgement bias, IMEs will be reported separately and not as a subgroup of SAEs. They will be processed in the same way as SAEs. Every aspect described for SAEs (including trial objectives and endpoints) also applies to an IME.

Severe local reactions can include severe pain, inflammation, induration and edema in the injection area[25]. Very rarely, abscess at injection site within 7 days of vaccine administration[26]. Nodules at the injection site with more than 2.5 centimeters in diameter and cellulitis[27].

11.2 CAUSALITY ASSESSMENT

The investigator(s) are obligated to assess the causal association between investigational vaccine and the occurrence of each SAE and IME and severe local reactions. The investigator(s) will use clinical judgement to determine the causal association. Alternative plausible causes, such as natural history of the underlying diseases, concomitant therapy, other risk factors, and the temporal causal association of the event to the investigational vaccine will be considered and investigated. The investigator(s) will also consult the Investigator’s Brochure to determine their assessment.

Causality should be assessed by the investigator(s) using AEFI causality algorithm developed by WHO for individual AEFI evaluation. When appropriate information is available the investigator(s) should arrive to the following possible conclusions:

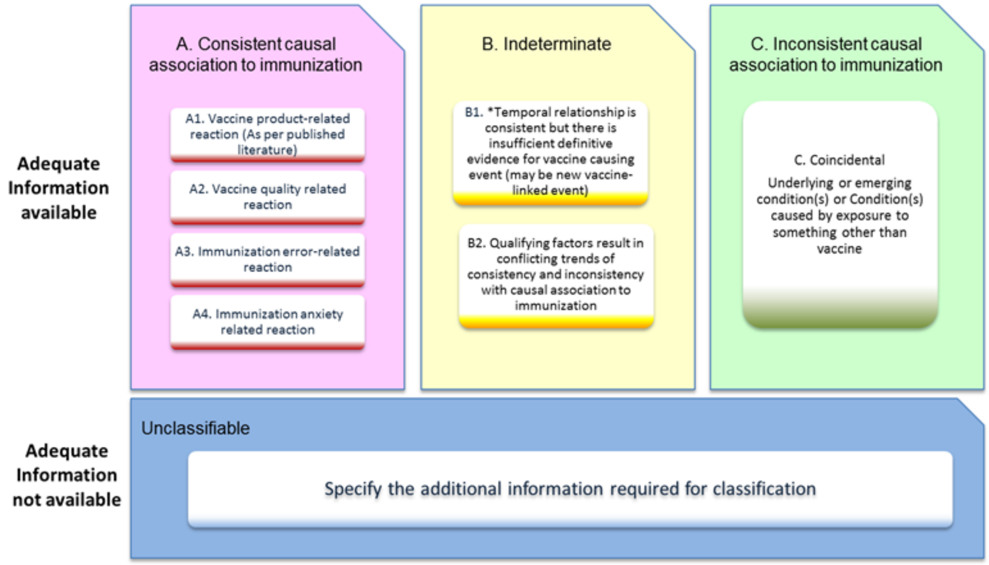

*B1 : Potential signal and maybe considered for investigation

If an event meets the criteria to be determined as ‘serious’ (see Section 11.1.1), additional examinations/tests will be performed by the investigator(s) in order to determine ALL possible contributing factors for each SAE.

Possible contributing factors include:

- Medical history.
- Other medication.
- Protocol required procedure.
- Other procedure not required by the protocol.
- Lack of efficacy of the vaccine, if applicable.
- Erroneous administration.
- Other cause (specify).

11.3 ACTION TAKEN REGARDING THE STUDY VACCINE

The action taken towards the study vaccine must be described as follows:

- Permanently discontinued.
- Stopped temporarily.
- No action taken.
- Not applicable.

11.4 OUTCOME

The outcome of each SAE/IME must be rated as follows:

- Recovered/resolved.
- Recovering/resolving.
- Not recovered/not resolved.
- Recovered with sequelae/resolved with sequelae.
- Fatal (SAEs only).

11.5 RECORDING OF SERIOUS ADVERSE EVENTS

All SAEs, IMEs and severe local reactions occurring during the clinical investigation must be documented in the source documents and on the SAE forms of the eCRF. The Investigator(s) will inquire about the occurrence of SAEs at every visit/contact during the study.

Whenever possible, diagnoses should be given when signs and symptoms are due to a common etiology (e.g., cough, runny nose, sneezing, sore throat, and head congestion should be reported as “upper respiratory infection”). Investigators must record their opinion concerning the possible causality of the SAE to the study vaccine in the source documents and on the eCRF. All measures required for SAEs management must be recorded in the source documents and reported according to Sponsor’s instructions.

All SAEs occurring at any time during the study will be followed by the Investigator(s) until satisfactory resolution (e.g., value back to baseline value) or stabilization or until final database lock. If necessary, in order to obtain additional information to ensure safety to the infant, additional blood and urine samples may be taken at the discretion of the Investigator(s). Certain long-term SAEs related to therapy cannot be followed until resolution within the setting of this study. In these cases, follow-up will be the responsibility of the treating physician.

11.6 REPORTING OF SERIOUS ADVERSE EVENTS AND IMPORTANT MEDICAL EVENTS TO THE SPONSOR

All SAEs, IMEs and severe local reactions, independent of the circumstances or suspected cause must be reported on a SAE Form by the Investigator(s) to the Sponsor within 24 h of their knowledge of the event, preferably by fax.

The SAE form should include a clearly written narrative describing signs, symptoms, and treatment of the event, diagnostic procedures, as well as any relevant laboratory data and

any sequelae, in order to allow a complete medical assessment of the case and independent determination of the possible causality.

Follow-up and outcomes should be reported for all infants who experience an SAE.

It is critical that the information provided on the SAE Form matches the information recorded in the source documents and on the eCRF for the same event.

Copies of additional laboratory test results, consultation reports, postmortem reports, hospital case reports, autopsy reports and other documents should be sent when requested and applicable. Follow-up reports relative to the infant's subsequent course must be submitted to the Sponsor until the event has subsided or, in the event of permanent impairment, until the condition stabilizes.

11.7 REPORTING OF SERIOUS ADVERSE EVENTS TO COMPETENT AUTHORITY/ETHICS COMMITTEES

FIDEC assumes responsibility for appropriate reporting of AEs to the regulatory authority in Uruguay. FIDEC will also report to the Investigator(s) all SAEs that are unlisted (unexpected) and associated with the use of the vaccine. The Investigator(s) must report these events to the appropriate Independent Ethics Committee/Institutional Review Board (IEC/IRB) that approved the protocol, unless otherwise required and documented by the IEC/IRB.

Adverse event reporting, including suspected unexpected serious adverse reactions (SUSARs), will be carried out in accordance with applicable local regulations.

After termination of the clinical study (determined as the last subject's last visit [LSLV]), any unexpected safety issue that changes the risk-benefit analysis and is likely to have an impact on the infants who have participated in the study, together with proposed actions, will be reported by the Sponsor to the competent authorities concerned as soon as possible.

12. ETHICAL ASPECTS

12.1 STUDY-SPECIFIC DESIGN CONSIDERATIONS

The parent(s)/legal representative of potential subjects will be fully informed of the nature of the study and of the risks and requirements of the study before any study-related assessment will be carried out. During the study, parent(s)/legal representative of the infant will be given any new information that may affect their decision to continue their child participating. They will be informed that their child's participation in the study is voluntary and that they may withdraw their child from the study at any time with no reason given, and without penalty or loss of benefits to which they or their child would otherwise be entitled. Only infants whose parent(s)/legal representative are fully able to understand the risks, benefits, and potential AEs of the study and who provide their consent voluntarily will be enrolled in the study.

12.2 REGULATORY ETHICS COMPLIANCE

12.2.1 *Investigator Responsibilities*

The Investigator(s) should be qualified by education, training, and experience to assume responsibility for the proper conduct of the study, should meet all the qualifications specified by the applicable regulatory requirements, and should provide evidence of such qualifications through up-to-date curriculum vitae or other relevant documentation requested by the Sponsor, the IRB/IEC, or the regulatory authorities.

The Investigator(s) are responsible for ensuring that the clinical study is performed in accordance with the protocol, current International Conference on Harmonization (ICH) guidelines on Good Clinical Practice (GCP), and applicable regulatory and country-specific requirements.

Good Clinical Practice is an international ethical and scientific quality standard for designing, conducting, recording, and reporting studies that involve the participation of human subjects. Compliance with this standard provides public assurance that the rights, safety, and well-being of study subjects are protected, consistent with the principles originating from the Declaration of Helsinki (1964 and revisions), and that the clinical study data are credible.

12.2.2 *Independent Ethics Committee or Institutional Review Board (IEC/IRB)*

An IRB/IEC should safeguard the rights, safety, and well-being of all study subjects in each country. Special attention should be paid to studies that may include vulnerable infants.

Before the start of the study, the Investigator(s) (or Sponsor where required) will provide the IEC/IRB with current and complete copies of the following documents:

- Final protocol and, if applicable, amendments.
- Sponsor-approved ICF (and any updates or any other written materials to be provided to the subjects).
- Sponsor-approved subject recruiting materials.
- Prescribing information of the licensed vaccine.

- Information on compensation for study-related injuries or payment to subjects for participation in the study, if applicable.
- Investigator's current curriculum vitae or other documentation evidencing qualifications (unless not required, as documented by the IEC/IRB).
- Information regarding funding, name of the Sponsor, institutional affiliations, other potential conflicts of interest, and incentives for subjects.
- Any other documents that the IEC/IRB may require to fulfill its obligation.

This study will be undertaken only after the IEC/IRB has given full written approval of the final protocol and amendments (if any), the ICF(s) and updates (if any), applicable recruiting materials, and any other written information to be provided to the subjects, and the Sponsor has received a copy of this approval. This approval letter must be dated and must clearly identify the IEC/IRB and the documents being approved.

During the study, the Investigator(s) (or Sponsor where required) will send the following documents and updates to the IEC/IRB for its review and approval, where appropriate:

- Protocol amendments.
- Revisions to the ICF and any other written materials to be provided to the infants' parents.
- New or revised subject recruiting materials approved by the Sponsor.
- Revisions to compensation for study-related injuries or payment to subjects or their parent(s)/legal representative for participation in the study.
- Prescribing information of the licensed vaccine.
- Summaries of the status of the study at intervals stipulated in guidelines of the IEC/IRB (at least annually).
- Reports of AEs that are serious, unlisted, and associated with the investigational product (SUSARs).
- New information that may adversely affect the safety of the infants or the conduct of the study.
- Deviations from or changes to the protocol to eliminate immediate hazards to the infants.
- Report of death of any infants under the Investigators' care.
- Notification if a new Investigator is responsible for the study at the clinical site.
- Development Safety Update Report, Short-Term Study Specific Safety Summary and Line Listings, where applicable.
- Any other requirements of the IEC/IRB.

For all protocol amendments (excluding the ones that are purely administrative, with no consequences for subjects, data or study conduct), the amendment and applicable ICF revisions must be submitted promptly to the IEC/IRB for review and approval before implementation of the change(s), except when necessary to eliminate immediate hazard to the study subjects. If a deviation from or a change to the protocol was implemented to eliminate an immediate hazard to study subjects, then the implemented deviation or

change, the reasons for it, and, if appropriate, the protocol amendment should be submitted to the IEC/IRB as soon as possible.

The Investigator(s) (or Sponsor where required) will notify the IEC/IRB about the study completion within 90 days after the end of the study (defined as LSLV).

12.2.3 *Informed Consent*

The parent(s)/legal representative of each infant must give written consent according to local requirements after the nature of the study has been fully explained. The consent form must be signed before performance of any study-related activity. The consent form that is used must be approved by both the Sponsor and the reviewing IEC/IRB. The informed consent should be in accordance with the principles that originated in the Declaration of Helsinki, current ICH and GCP guidelines, applicable regulatory requirements, and Sponsor policy.

Before enrollment in the study, the Investigator(s) or an authorized member of the clinical staff must explain to the parent(s)/legal representative of potential subjects the aims, methods, reasonably anticipated benefits, and potential hazards of the study, and any discomfort participation in the study may entail. Infants' parent(s)/legal representative will be informed that the infant's participation is voluntary and that they may refuse to allow the infant to participate or withdraw consent for the infant to participate at any time, without penalty or loss of benefits to which the parent(s)/legal representative and/or infant was entitled. Finally, they will be told that the Investigator(s) will maintain a subject identification register for the purposes of long-term follow-up if needed and that the infant's records may be accessed by health authorities and authorized Sponsor staff without violating the confidentiality of the infant, to the extent permitted by the applicable law(s) or regulations. By signing the ICF the infant's parent(s)/legal representative is authorizing such access, and agrees to allow the infant's study physician to re-contact the infant's parent(s)/legal representative for the purpose of obtaining consent for additional safety evaluations, if needed.

The ICF will include a paragraph whereby the infant's parent(s)/legal representative allow or not the use of the infant's biological samples for additional polio related research, if needed.

The language about the study used in the oral and written information, including the ICF, should be non-technical and practical and should be understandable to the infants' parent(s)/legal representative. The infants' parent(s)/legal representative will be given sufficient time to read the ICF and the opportunity to ask questions. After this explanation and before entry of the infant into the study, consent should be appropriately recorded by means of the infant's parent(s)/legal representative personally dated signature. After having obtained consent, a copy of the ICF must be given to the infant's parent(s)/legal representative.

If a parent(s)/legal representative of an infant is unable to read or write, an impartial witness should be present for the entire informed consent process (which includes reading and explaining all written information) and should personally date and sign the ICF after their oral consent is obtained, if permitted by local law.

12.2.4 *Privacy of Personal Data*

The collection and processing of personal data from infants enrolled in the study will be limited to those data that are necessary to investigate the safety, quality and immunogenicity of the vaccine used in the study.

These data must be collected and processed with adequate precautions to ensure confidentiality and compliance with applicable data privacy protection laws and regulations. Appropriate technical and organizational measures to protect the personal data against unauthorized disclosures or access, accidental or unlawful destruction, or accidental loss or alteration must be put in place. Sponsor personnel whose responsibilities require access to personal data need to agree to keep the identity of the study subjects confidential.

The informed consent obtained from the infants' parent(s)/legal representative includes explicit consent for the processing of personal data and for the Investigator(s) to allow direct access to infants' original medical records for study-related monitoring, audit, IEC/IRB review, and regulatory inspection. This consent also addresses the transfer of the data to other entities and to other countries.

13. ADMINISTRATIVE REQUIREMENTS

13.1 PROTOCOL AMENDMENTS/NOTIFICATIONS

Neither the Investigator(s) nor the Sponsor will modify this protocol without a formal amendment (except modifications that do not alter the benefit/risk-see next paragraph). All protocol amendments must be issued by the Sponsor and signed and dated by the Investigator(s). Protocol amendments must not be implemented without prior IEC/IRB approval nor when the relevant competent authority has raised any grounds for non-acceptance, except when necessary to eliminate immediate hazard to the infants, in which case an amendment must be promptly submitted to the IEC/IRB and relevant competent authority. Documentation of amendment approval by the Investigator(s) and IEC/IRB must be provided to the Sponsor or its designee.

When the change(s) involves only logistic or administrative aspects of the study, the IRB (and IEC where required) only needs to be notified.

13.2 SUBJECT IDENTIFICATION AND ENROLLMENT LOGS

The Investigator(s) agree to complete a subject identification and enrollment log to permit easy identification of each infant during and after the study. This document will be reviewed by the monitor for completeness.

The subject identification and enrollment log will be treated as confidential and will be filed by the Investigator(s) in the study file. To ensure subject confidentiality, no copies will be made. All reports and communications related to the study will identify infants by initials and/or assigned number only.

13.3 SOURCE DOCUMENTATION

At a minimum, source documentation must be available for the following to confirm data collected in the eCRF: subject identification, eligibility, and study identification; study discussion and date of informed consent, dates of visits, results of safety and immunogenicity parameters as required by the protocol, record of all AEs, follow-up of AEs, concomitant medication, study vaccine receipt/dispensing/return records, study vaccine administration information, laboratory printouts, date of study completion, and reason for early discontinuation of study vaccine or withdrawal from the study, if applicable.

Direct access to source documentation (medical records) must be allowed for the purpose of verifying that the data recorded on the eCRF are consistent with the original source data.

It is recommended that the author of an entry in the source documents be identifiable.

At a minimum, the type and level of detail of source data available for a study subject should be consistent with that commonly recorded at the clinical site as a basis for standard medical care. Specific details required as source data for the study will be reviewed with the Investigator(s) before the study and will be described in the monitoring guidelines (or other equivalent document). The nature and location of all source documents will be identified in the Source Document Identification Form. Data that will be recorded directly into the eCRF.

13.4 CASE REPORT FORM COMPLETION

Electronic Data Capture (EDC) will be used for this study. The study data will be transcribed by study personnel from the source documents into an eCRF, and transmitted in a secure manner to the Sponsor. The electronic file will be considered to be the eCRF.

All eCRF entries, corrections, and alterations must be made by the Investigator(s) or other authorized study-site personnel.

Worksheets may be used for the capture of some data to facilitate completion of the eCRF. Any such worksheet will become part of the infant's source documentation. Such worksheet should not resemble an eCRF. All data related to the study must be recorded on the eCRFs prepared by the Sponsor. Data must be entered into the eCRFs in English. Designated site personnel must complete the eCRF as soon as possible after a subject visit, and the forms should be available for review at the next scheduled monitoring visit.

The Investigator(s) must verify that all data entries on the eCRFs are accurate and correct.

13.5 MONITORING

The monitoring of the study will be done under the responsibility of the Sponsor by VaxTrials.

The monitor will perform sites evaluations, study initiation, during study and study closure on-site visits as frequently as necessary. The monitor will record the dates of the visits in a study site visit log that will be kept at the clinical site. The first post-initiation visit will be made as soon as possible after enrollment has begun. At these visits, the monitor will compare the data entered into the eCRFs with the hospital or clinic records (source documents). The nature and location of all source documents will be identified to ensure that all sources of original data required to complete the eCRF are known to the Sponsor and clinical staff and are accessible for verification by the Sponsor site contact. If electronic records are maintained at the investigational site, the method of verification must be discussed with the clinical staff.

Direct access to source documentation (medical records) must be allowed at all times for the purpose of verifying that the data recorded in the eCRF are consistent with the original source data. Findings from this review of eCRFs and source documents will be discussed with the clinical staff. During on-site monitoring visits (notified and agreed upfront with the clinical staff), the relevant clinical staff will be available, the source documentation will be accessible, and a suitable environment for review of study-related documents will be provided. The monitor will meet with the Investigator(s) on a regular basis during the study to provide feedback on the study conduct.

13.6 DATA MANAGEMENT

Data management of the study will be performed under the responsibility of the Sponsor by VaxTrials, who will subcontract this service and the eCRF management.

After the monitor reviews the data entered into the eCRFs for completeness and accuracy and the data are released by the Investigator(s), data will be uploaded into the clinical database to perform cleaning activities. Computerized data cleaning checks will be used in addition to manual review, including listings review, to check for discrepancies and to ensure consistency and completeness of the data.

If necessary, queries will be generated in the EDC tool. The Investigator(s) or an authorized member of the clinical staff must adjust the eCRF (if applicable) and complete the query. If corrections to an eCRF are needed after the initial entry into the eCRF, this can be done in 3 different ways: 1- site personnel can make corrections in the EDC tool at their own initiative or as a response to an auto query (generated by the EDC tool), 2- the site manager can generate a query (field data correction form [DCF]) for resolution by the clinical staff, and 3- the clinical data manager can generate a query for resolution by the clinical staff.

The clinical database will be locked as soon as it is considered clean. Only authorized and well-documented updates to the study data are possible after database lock. The locked database is used in the final statistical analysis for study reporting. Measures will be undertaken to protect subject data handed over by the Investigator(s) to the data management department and during inspections against disclosure to unauthorized third parties. Subject confidentiality will be maintained at all times.

13.7 DATA QUALITY ASSURANCE

The accuracy and reliability of the study data will be assured by the selection of qualified Investigators and appropriate study sites, review of protocol procedures with the Investigator(s) and associated personnel prior to the study, and by periodic monitoring visits by the Sponsor or designate.

The Sponsor or his designee will review the eCRF system for accuracy and completeness during (on-site) monitoring visits and after transmission to the Sponsor; any discrepancies will be resolved with the Investigator(s) or designee, as appropriate. After upload of the data into the clinical study database, their accuracy verified using appropriate validation programs.

In accordance with Good Clinical Research Practice Guidelines and Recommendations, the Sponsor will be entitled to audit the facilities used in the clinical and laboratory parts of the study, as well as to access all the data files pertaining to the study. Similar procedures may also be conducted by agents of any regulatory body, either as part of a national GCP compliance program or to review the results of this study in support of a regulatory submission. The Investigator(s) should immediately notify the Sponsor if they have been contacted by a regulatory agency concerning an upcoming inspection.

13.8 ON-SITE AUDITS

Representatives of the Sponsor’s clinical quality assurance department or any other qualified auditor appointed by the Sponsor may visit the clinical site at any time during or after completion of the study to conduct an audit of the study in compliance with regulatory guidelines and company policy. These audits will require access to all study records, including source documents, for inspection and comparison with the eCRFs. Subject privacy must, however, be respected. The Investigator(s) and clinical staff are to be present and available for consultation during routinely scheduled site audit visits conducted by the Sponsor or its designees.

Similar procedures may also be conducted by agents of any regulatory body, either as part of a national GCP compliance program or to review the results of this study in support of a regulatory submission. The Investigator(s) should immediately notify the Sponsor if they have been contacted by a regulatory agency concerning an upcoming inspection.

13.9 STUDY TERMINATION

The Sponsor has the right to terminate the study at any time. In the event of early termination of the study or temporary halt by the Sponsor, the IEC/IRB and the regulatory authorities should be notified within 15 calendar days and should be provided with a detailed written explanation of the reasons for the termination/halt.

An end-of-study declaration will be submitted to the regulatory authorities and IEC/IRB after the complete study has ended. This notification will be submitted within 90 days after the end of the study.

13.10 RECORD RETENTION

In compliance with the ICH/GCP guidelines, the Investigator(s)/Institution will maintain all eCRFs and all source documents that support the data collected from each infant, as well as all study documents as specified in ICH/GCP Section 8, Essential Documents for the Conduct of a Clinical Trial, and all study documents as specified by the applicable regulatory requirement(s). The Investigator(s)/Institution will take measures to prevent accidental or premature destruction of these documents.

Essential documents must be retained until at least 15 years after the last approval of a marketing application in an ICH region and until there are no pending or contemplated marketing applications in an ICH region or until at least 5 years have elapsed since the formal discontinuation of clinical development of the IMP. These documents will be retained for a longer period if required according to the applicable regulatory requirements or per agreement with the Sponsor. It is the responsibility of the Sponsor to inform the Investigator(s)/Institution as to when these documents no longer need to be retained.

If the responsible Investigator(s) retires, relocates, or for any other reasons withdraws from his responsibility of keeping the study records, custody must be transferred to a person who will accept the responsibility. The Sponsor must be notified in writing of the name and address of the new custodian. Under no circumstance shall the Investigator(s) relocate or dispose of any study documents without having obtained written approval from the Sponsor.

If it becomes necessary for the Sponsor or the appropriate regulatory authority to review any documentation related to the study, the Investigator(s) must permit access to such reports.

13.11 USE OF INFORMATION AND PUBLICATION

All information, including but not limited to, information regarding the study vaccine or the Sponsor's operations (e.g., patent application, formulas, manufacturing processes, basic scientific data, prior clinical data, formulation information) supplied by the Sponsor to the Investigator(s) and not previously published, and any data generated as a result of this study are considered confidential and remain the sole property of the Sponsor. The Investigator(s) agrees to maintain this information in confidence, to use this information only to accomplish this study, and not to use it for other purposes without the Sponsor's prior written consent.

The Investigator(s) understands that the information generated in this clinical study will be used by the Sponsor in connection with the continued development of the study vaccine, and thus may be disclosed as required to other clinical Investigators or regulatory agencies.

To permit information derived from the clinical studies to be used, the Investigator(s) is obliged to provide the Sponsor with all data obtained in the study.

The results of the study will be reported in a Clinical Study Report generated under the responsibility of the Sponsor and will contain eCRF data from all clinical sites that participated in the study. Recruitment performance or specific expertise related to the nature and the key assessment parameters of the study will be used to determine a coordinating Investigator.

Clinical narratives will be written for the following events (for example):

- All deaths (irrespective of causal association to vaccine).
- All other SAEs and IMEs after vaccination.
- All discontinuations of the study vaccine due to AEs (irrespective of causal association to vaccine).
- At the discretion of the team and after statistical analysis of the data, certain discontinuations not related to AEs or treatment failure, i.e., related to lost to follow-up or withdrawal of consent (irrespective of treatment group).
- Any events of special interest explicitly requested by the regulatory agencies.

The coordinating Investigator(s) will sign off the final version of the Clinical Study Report. A summary of this final version will be provided to the Investigators, the applicable regulatory authorities, and the IECs/IRBs, if required by the applicable regulatory requirements, within 1 year after the end of the study (LSLV).

The Sponsor shall have the right to publish study data and information without approval from the Investigator(s). If an Investigator wishes to publish information from the study, a copy of the manuscript must be provided to the Sponsor for review at least 30 days before submission for publication or presentation. Expedited reviews will be arranged for abstracts, poster presentations, or other materials. If requested by the Sponsor in writing, the Investigator(s) will withhold such publication for up to an additional 30 days to allow for filing of a patent application. In the event that issues arise regarding scientific integrity or regulatory compliance, the Sponsor will review these issues with the Investigator(s). The Sponsor will not mandate modifications to scientific content and does not have the right to suppress information. Authorship of publications resulting from this study will be based on the guidelines on authorship, such as those described in the Recommendations for the Conduct, Reporting, Editing, and Publication of Scholarly Work in Medical Journals, which state that the named authors must have made a significant contribution to the design of the study or analysis and interpretation of the data, provided critical review of the paper, and given final approval of the final version.

13.12 REGISTRATION OF CLINICAL STUDIES AND DISCLOSURE OF RESULTS

The Sponsor will register the existence and disclose the results of this clinical study as required by law, on Clinicaltrials.gov and the WHO International Clinical Trials Registry Platform (ICTRP).

For the public disclosure of clinical study documentation or data, e.g., the study protocol or clinical study report, appropriate measures will be taken to redact such material so as to

protect the privacy and confidentiality of the data as applicable to the study subjects in agreement with the legislative authority requiring such disclosure.

13.13 INVESTIGATOR INDEMNITY

The Sponsor holds and will maintain an adequate insurance policy covering damages arising out of FIDEC-sponsored clinical research studies.

The Sponsor will indemnify the Investigator(s) and hold them harmless for claims related to damages arising from the investigation, provided that the study vaccine was administered under the Investigator(s) or deputy's supervision and in strict accordance with accepted medical practice and the study protocol.

The Investigator(s) must notify the Sponsor immediately upon notice of any claims or lawsuits.

13.14 CONFIDENTIALITY

All study documents are provided by the Sponsor to the Investigator(s) and appointed clinical staff in confidence. None of this material may be disclosed to any party not directly involved in the study without the Sponsor's written permission.

The Investigator(s) must assure that infants' anonymity will be maintained. The Investigator(s) will keep a separate list with at least the initials, the subjects' study numbers, names, addresses, and telephone numbers. The Investigator(s) will maintain this for the longest period of time allowed by his/her own institution and, in any case, until further communication from the Sponsor.

14. REFERENCES

1. Global Polio Eradication Initiative. Polio eradication and endgame strategic plan 2013-2018. Available at http://polioeradication.org/who-we-are/strategy/ Last accesses 20/07/2017

2. Global Polio Eradication Initiative. Endemic Countries. Available at http://polioeradication.org/where-we-work/polio-endemic-countries/ Copyright 2016. Last accessed 20/07/2017.

3. Sabin, A B. "Oral Poliovirus Vaccine: History of Its Development and Use and Current Challenge to Eliminate Poliomyelitis from the World." The Journal of infectious diseases 151, no. 3 (1985): 420-3

4. Patriarca, P A, P F Wright, and T J John. "Factors Affecting the Immunogenicity of Oral Poliovirus Vaccine in Developing Countries: Review." Reviews of infectious diseases 13, no. 5 (1991): 926-39

5. O'Reilly, Kathleen M, Elias Durry, Obaid ul Islam, Arshad Quddus, Ni'ma Abid, Tahir P Mir, Rudi H Tangermann, R Bruce Aylward, and Nicholas C Grassly. "The Effect of Mass Immunisation Campaigns and New Oral Poliovirus Vaccines on the Incidence of Poliomyelitis in Pakistan and Afghanistan, 2001-11: A Retrospective Analysis." Lancet 380, no. 9840 (2012).

6. World Health Organization. Immunization, Vaccines and Biologicals. IPV introduction and RI strengthening. Available at: http://www.who.int/immunization/diseases/poliomyelitis/endgame_objective2/inactivated_polio_vaccine/en/ Last accessed 20/07/2017

7. O'Ryan M, Bandyopadhyay AS, Villena R, et al. Inactivated poliovirus vaccine given alone or in a sequential schedule with bivalent oral poliovirus vaccine in Chilean infants: a randomised, controlled, open-label, phase 4, non-inferiority study. Lancet Infect Dis 2015 : 15:1273–82.

8. Sáez-Llorens X, Clemens R, Leroux-Roels G, et al. Immunogenicity and safety of a novel monovalent high-dose inactivated poliovirus type 2 vaccine in infants: a comparative, observer-blind, randomised, controlled trial. Lancet Infect Dis 2016 : 16:321–30

9. Asturias EJ, Bandyopadhyay AS, Self S, et al. Humoral and intestinal immunity induced by new schedules of bivalent oral poliovirus vaccine and one or two doses of inactivated poliovirus vaccine in Latin American infants: an open-label randomised controlled trial. Lancet 2016; 388:158–69.

10. Global Polio Eradication Initiative. Semi-Annual status Report, July to December 2016. Available at http://polioeradication.org/wp-content/uploads/2017/04/Status-Report_Jul-Dec2016.pdf Last accessed 20/07/2017

11. Pan American Health Organization. TAG recommendations about the use of IPV. Available at: http://www.paho.org/hq/index.php?option=com_content&view=article&id=12134%3Atag-recommendations-ipv&catid=1875%3Apolio-highlights&Itemid=2244&lang=en
Last accessed: 20/07/2017

12. World Health Organization. Weekly epidemiological record. June 1, 2017. No 22, 2017, 301-320

13. Pan American Health Organization. Immunization Newsletter. Volume XXXVIII Number 2. Copyright June 2016.

14. Vidor E et al. Poliovirus Vaccine Inactivated. In Plotkin SA, Orenstein WA, Offit PA. Vaccines, 2013, 6th edition 2013. Philadelphia: Elsevier-Saunders, pp. 573–597

15. Iqbal S et al. Preparation for Global Introduction of Inactivated Poliovirus Vaccine: Safety Evidence from the US Vaccine Adverse Event Reporting System, 2000–12. Lancet Infect Dis 2015. Volume 15, No. 10, p1175–1182, October 2015.

16. Resik S et al. Priming after a fractional dose of inactivated poliovirus vaccine. The New England Journal of Medicine 2013; 368:416-24

17. Anand A et al. Early priming with inactivated poliovirus vaccine (IPV) and intradermal fractional dose IPV administered by a microneedle device: A randomized controlled trial. Vaccine 2015; 33(48):6816-22.

18. Grassly N. Immunogenicity and Effectiveness of Routine Immunization With 1 or 2 Doses of Inactivated Poliovirus Vaccine: Systematic Review and Meta-analysis. The Journal of Infection Diseases 2014; doi: 10.1093/infdis/jit601

19. Resik S et al. Randomized Controlled Clinical Trial of Fractional Doses of Inactivated Poliovirus Vaccine Administered Intradermally by Needle-Free Device in Cuba. The Journal of Infectious Diseases 2010; 201(9):1344–1352

20. World Health Organization. Polio Vaccines: WHO Position Paper. No. 12, 2016, 91. 145-168. Available at: http://www.who.int/wer/2016/wer9112.pdf?ua=1 Last accessed 20/07/2017

21. Anand Abhijeet, et al. Immunogenicity to poliovirus type 2 following two doses of fractional intradermal inactivates poliovirus vaccine: A novel dose sparing immunization Schedule. Vaccine. 2017. May 19;34 (22): 2993-2998.

22. Resik S et al. Immune responses after fractional doses of inactivated poliovirus vaccine using newly developed intradermal jet injectors: A randomized controlled trial in Cuba. Vaccine 2015; 33 (2) :307-313

23. World Health Organization. Immunization, Vaccines and Biologicals. Fractional dose IPV. Available at

http://www.who.int/immunization/diseases/poliomyelitis/endgame_objective2/inactivated_polio_vaccine/fractional_dose/en/ Last accessed: 20/07/2017

24. Weldon WC, Oberste MS, Pallansch MA, Standardized Methods for Detection of Poliovirus Antibodies. Poliovirus: Methods and protocols, pp 145-176. 2016; DOI 10.1007/978-1-4939-3292-4_8; Online ISBN 978-1-4939-3292-4

25. Gidudu J et al. A local reaction at or near injection site: case definition and guidelines of data collection, analysis and presentation of immunization safety data. Vaccine (2008), Vol.26: 6800-6831

26. Kohl K et al. Abscess at injection site: case definition and guidelines of data collection, analysis and presentation of immunization safety data. Vaccine (2007), Vol.25: 5821-5838

27. Halperin S et al. Cellulitis at injection site: case definition and guidelines of data collection, analysis and presentation of immunization safety data. Vaccine (2007), Vol.25: 5803-5820
